# Rational Design, Synthesis, Biological Evaluation, and In Silico Study of *N*‐Cinnamamide/Benzamide‐(4‐(Substituted Phenyl)‐6‐(4‐Cyclohexylphenyl)pyrimidin‐2‐yl) Derivatives as EGFR Inhibitors Targeting EGFR^WT^ , EGFR^T790M^ , and EGFR^L858R^ ^/T790M/C797S^ in NSCLC

**DOI:** 10.1111/cbdd.70390

**Published:** 2026-09-08

**Authors:** Rohit Pal, Gurubasavaraja Swamy Purawarga Matada, Ghanshyam Teli, Manjushree BV, Anguraj Moulishankar, Viney Chawla, Pooja A. Chawla

**Affiliations:** ^1^ Department of Pharmaceutical Chemistry, Integrated Drug Discovery Centre Acharya & BM Reddy College of Pharmacy Bengaluru Karnataka India; ^2^ School of Pharmacy Sangam University Bhilwara Rajasthan India; ^3^ University Institute of Pharmaceutical Sciences and Research, Baba Farid University of Health Sciences Faridkot Punjab India

**Keywords:** ADMET, docking, EGFR, lung cancer, pyrimidine

## Abstract

Non‐small cell lung cancer (NSCLC), driven by mutations in the epidermal growth factor receptor (EGFR), presents significant therapeutic challenges, particularly due to drug resistance. This study describes the design, synthesis, and evaluation of a series of novel *N*‐Cinnamamide/benzamide‐(4‐(substituted phenyl)‐6‐(4‐cyclohexylphenyl)pyrimidin‐2‐yl) derivatives (**Ri–Rxii**) as EGFR inhibitors. Among these, compound **Riv** exhibited superior antiproliferative activity against wild‐type and mutant EGFR‐driven NSCLC cell lines, including EGFR^L858R/T790M^ and the drug‐resistant EGFR^L858R/T790M/C797S^ variant, with low IC_50_ values and minimal cytotoxicity towards normal cell lines. Compound **Riv** effectively induced cell cycle arrest at the S and G2/M phases and triggered apoptosis, with 54.38% of cells undergoing apoptotic or necrotic death. Additionally, **Riv** exhibited strong antioxidant activity, outperforming the standard ascorbic acid in DPPH and ABTS assays, indicating its ability to reduce oxidative stress. Molecular docking and dynamic simulation studies confirmed stable binding interactions with key EGFR residues, and DFT analysis revealed favorable electronic properties. Furthermore, in silico ADMET predictions highlighted **Riv**'s promising pharmacokinetic and toxicity profiles, supporting its potential as a safe and effective anticancer agent. This comprehensive evaluation positions **Riv** as a strong candidate for overcoming drug resistance in NSCLC treatment.

## Graphical abstract

Rational Design, Synthesis, Biological Evaluation, and In Silico Investigation of N‐Cinnamamide/Benzamide‐(4‐(Substituted Phenyl)‐6‐(4‐Cyclohexylphenyl)pyrimidin‐2‐yl) Derivatives as EGFR Inhibitors Targeting Wild‐Type and Mutant EGFR in NSCLC.

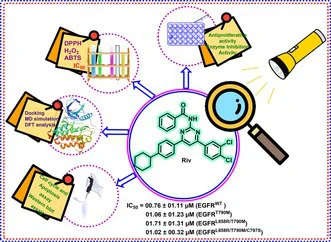

## 1 Introduction

**FIGURE 1:**
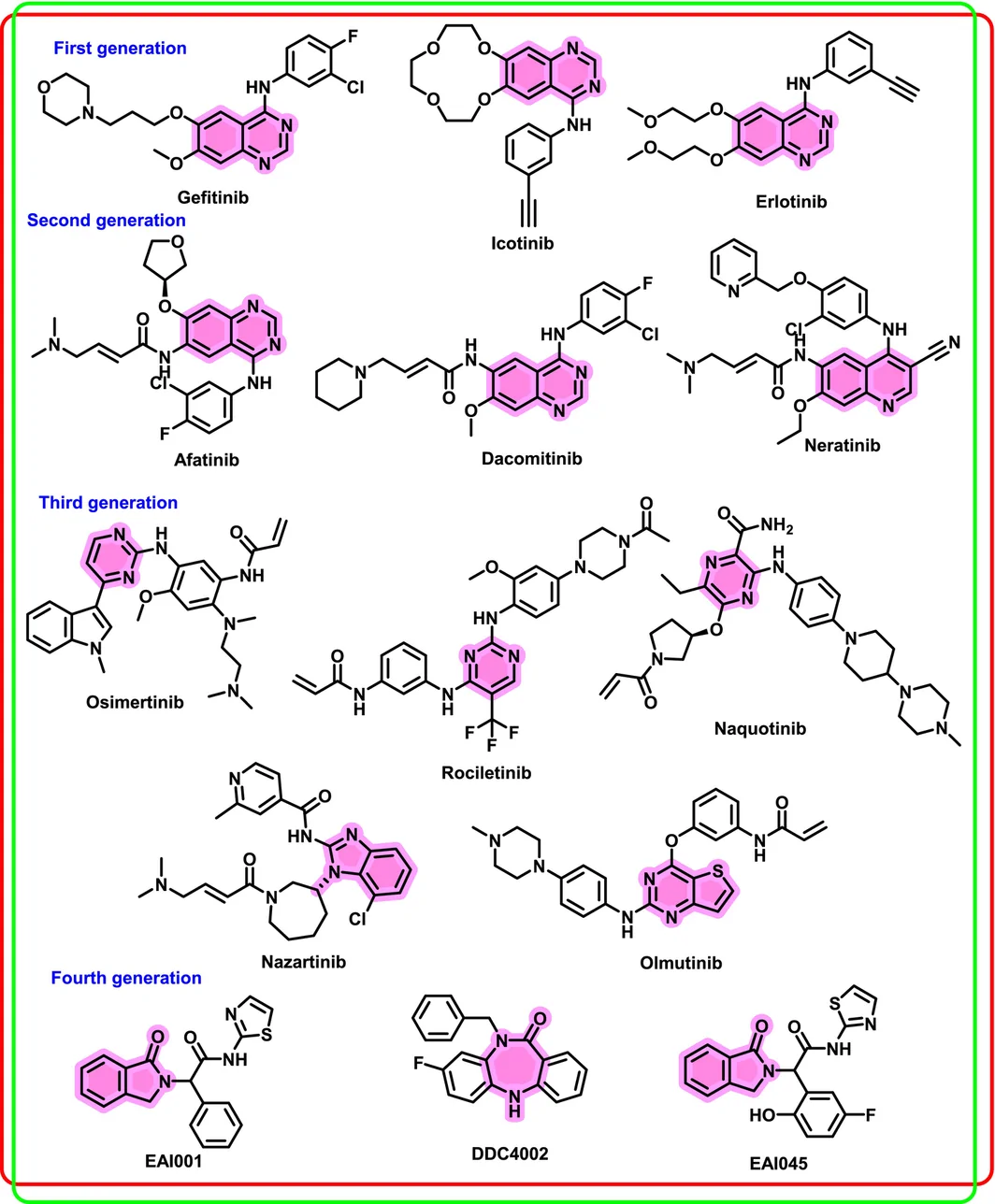
Different generation of epidermal growth factor receptor‐inhibitors (EGFRIs).

Lung cancer remains a leading cause of cancer‐related deaths worldwide. Significant advances in treatment, particularly in targeted therapies, have led to substantial improvements in survival rates (Pal et al. 2023b). Lung cancer is broadly categorized into non‐small cell lung cancer (NSCLC) and small cell lung cancer (SCLC) (Ayati et al. 2020). Epidermal Growth Factor Receptor (EGFR) is one of the most important enzymes in the tyrosine kinase family that triggers NSCLC. EGFR is typically a transmembrane receptor located on the cell surface. Based on structural similarities, EGFR is categorized as ErbB1 (HER1), ErbB2 (HER2, neu in rodents), ErbB3 (HER3), and ErbB4 (HER4), all of which are activated upon ligand binding. Adverse circumstances can result from overexpression of EGFR, primarily in glioblastoma, breast cancer, and lung cancer. When epidermal growth factor pathways are activated, neo‐angiogenesis, enhanced metastatic potential, and cancer proliferation begin (Hosamani et al. 2024). Many downstream signaling pathways are triggered by the overexpression of EGFR, leading to more aggressive growth and invasive traits. The EGFR gene's activating mutations significantly influenced non‐small cell lung cancer (NSCLC) therapy approaches (Rossi et al. 2019; Marchetti et al. 2012). The recent identification of novel EGFR inhibitors was crucial in combating these mutations. Indeed, missense mutations have been occurring in EGFR enzymes. These mutations significantly affect the medications by exhibiting resistance (Choi et al. 2018). Because of the protein's altered actions, researchers have been compelled to develop new drugs that prevent and suppress resistance. Nucleotide alterations are used to classify EGFR mutations. Exon19 deletion was identified as the first mutation. Resistance was observed in medications such as erlotinib and gefitinib, which were widely used to treat lung cancer (Rossi et al. 2019). These drugs block the target enzyme and are regarded as first‐generation (Figure 1) (Pal, Teli, et al. 2024; Bello 2018). By altering the resident R‐groups, second‐generation medications were created using a similar quinazoline scaffold. Drugs such as neratinib and afatinib demonstrated possible suppression of the recurring mutations (Lu et al. 2020; Zhou et al. 2022). Everything was well until the wild‐type EGFR enzyme showed a novel missense change that replaced a specific AUG gene with a UAU gene. As a result, the amino acid threonine changed to methionine at position 790. Additionally, this new T790M mutation altered the binding property functions that were discovered to be toxicity‐related (Bello 2018; Pawara et al. 2021). A novel class of drugs with a structural modification from quinazoline to pyrimidine rings emerged to combat mutations and reported toxicity. Rocletinib and AZD9291, the most recent advancement in third‐generation compounds, demonstrated enhanced activity against mutations such as the T790M mutation and exon 19 deletion (Zhang, Zou, et al. 2018). Despite their effectiveness, the molecules had several disadvantages, including the creation of novel resistance in the form of a mutation in the C797S gene that changed the genetic coding. The protein showed functional and structural alterations as a result of the C797S mutation (Pal et al. 2023a; Pawara et al. 2022).

In many synthetic drugs, bioactive natural products, pharmaceuticals, and agrochemicals today, nitrogen is the primary heterocyclic molecule moiety (Singh et al. 2021). Heterocyclic compounds that include nitrogen have been essential in boosting the effectiveness of medications. The activity is increased by an active nitrogen atom's range of interactions (Pal, Matada, et al. 2024; Raghavendra et al. 2023). In medicinal chemistry literature, pyrimidine is an often‐encountered heterocycle with a broad range of pharmacologic properties, including antibacterial, anti‐inflammatory, antifungal, antimalarial, anti‐convulsant, anti‐hypertensive, anti‐HIV, antiviral, and anticancer activity (Patel et al. 2017; Shaikh et al. 2021; Lu 2023). Medicinal chemists have demonstrated various biological activities by applying different active groups to the pyrimidine molecule using various synthetic procedures. Therefore, we have designed novel 2‐amino pyrimidine derivatives. The pyrimidine core ensures strong binding to the hinge region via hydrogen bonds, mimicking ATP and enhancing binding specificity. Moreover, the pyrimidine ring often acts as a bio‐isostere for adenine, mimicking the purine base of ATP. This allows the compound to compete with ATP for binding at the kinase active site (Milik et al. 2017). Cyclohexyl tail extends into regions not occupied by ATP, engaging in hydrophobic interactions and enhancing the inhibitor's affinity for EGFR. Furthermore, substituents on the pyrimidine phenyl region (R_1_) are designed to improve selectivity and binding affinity by targeting specific pockets unique to EGFR. For example, bulky or polar groups can help differentiate between mutant and wild‐type EGFR. The R_2_ groups are engineered to occupy the solvent‐exposed region or interact with additional binding sites (e.g., hydrophobic pockets or polar residues). The benzyl or other extended groups can provide steric bulk, and hydrophobicity, or engage in π‐stacking interactions. This combination improves selectivity, potency, and pharmacokinetic properties, making it a robust scaffold for targeting both wild‐type and mutant EGFRs, especially in drug‐resistant cancers (Ayati et al. 2020).

### 1.1 Rational Design

**FIGURE 2:**
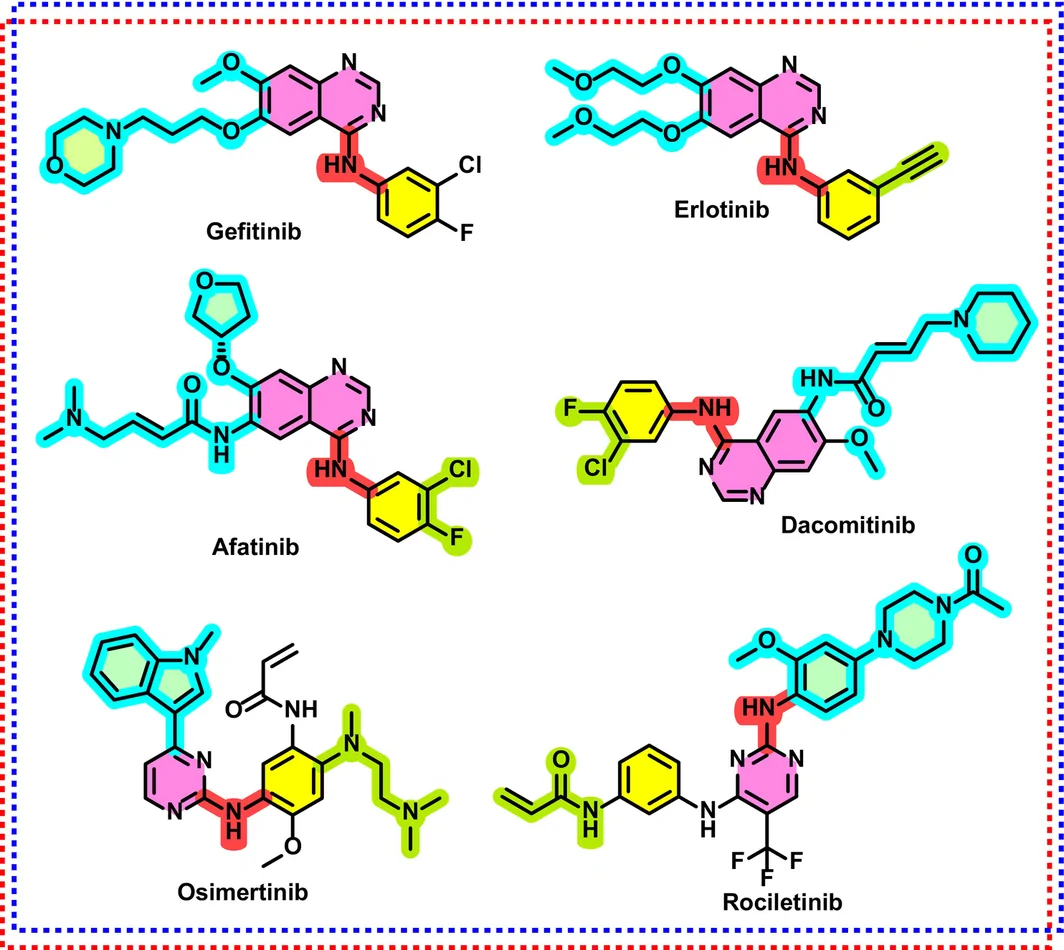
Basic pharmacophore features of EGFR inhibitors. (i) Central Core (Pink): The aromatic heterocycle is crucial for binding to the kinase active site, ensuring key interactions and maintaining selectivity. (ii) Hydrophobic Head (Cyan): Hydrophobic regions optimized for interaction with specific EGFR residues, including mutant or resistant forms. (iii) NH Spacer (Red): Essential for maintaining spatial flexibility and aligning functional groups for effective binding. (iv) Hydrophobic tail (Yellow): Functional groups (e.g., halogens, amino groups) enhancing polar and hydrophobic interactions for improved potency and selectivity.

**FIGURE 3:**
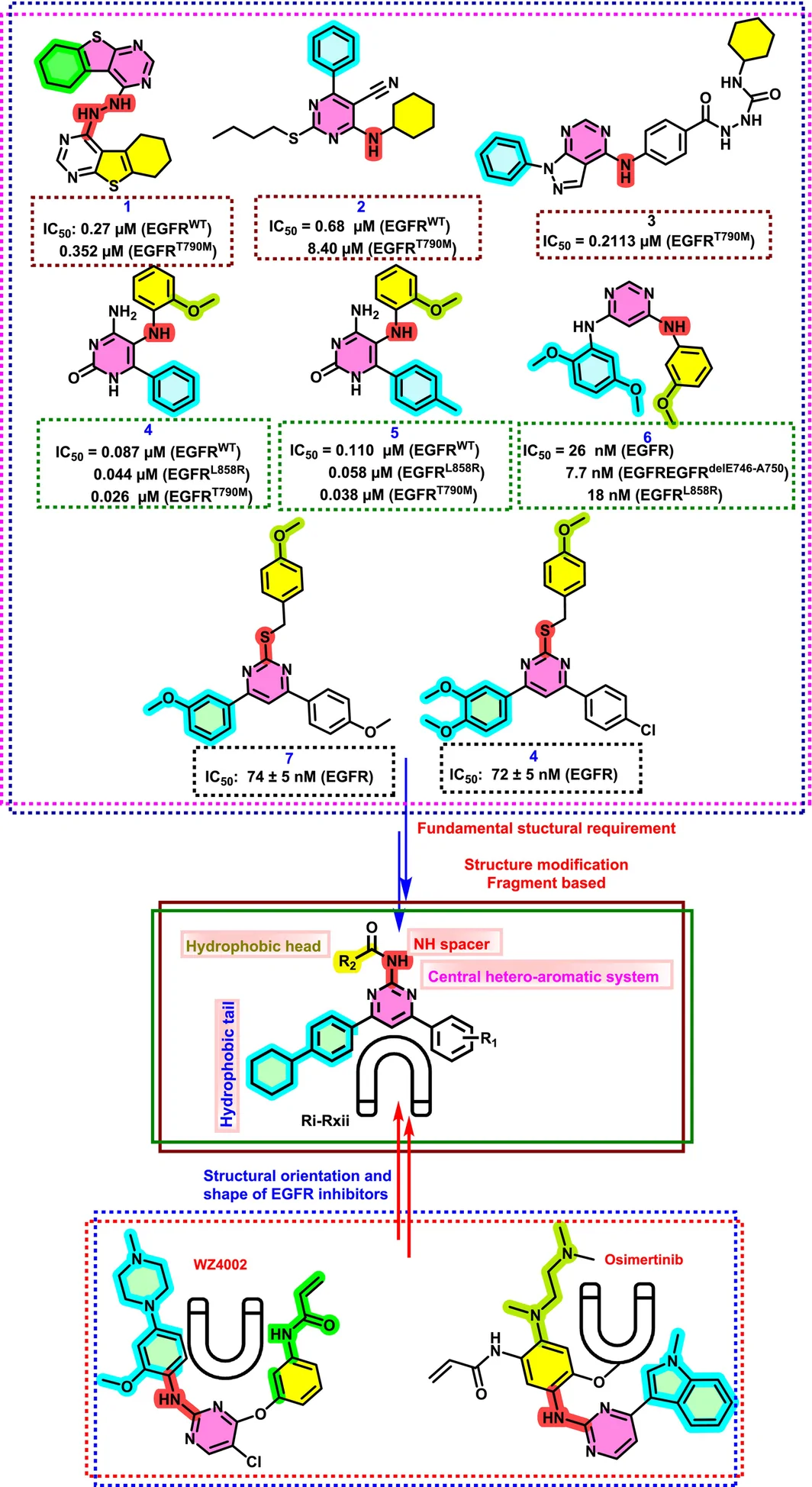
Structural insights and design strategies for EGFR inhibitors (**Ri‐Rxii**). Fragment‐based optimization showcases lead molecules with IC_50_ values and structural modifications, emphasizing the role of the pyrimidine, cyclohexyl group, and NH spacer for enhanced potency.

The basic structural forms of EGFR inhibitors are built around a few fundamental components tailored to interact with the EGFR tyrosine kinase domain effectively (Figure 2). These inhibitors are generally ATP‐competitive, meaning they compete with ATP to bind at the active site of EGFR, thereby blocking its signaling. Most EGFR inhibitors feature an aromatic or heteroaromatic core, which provides a planar structure crucial for π‐π stacking and hydrophobic interactions with the EGFR binding pocket. This core helps anchor the inhibitor in the ATP‐binding site (Shi et al. 2022). The hinge region is a critical part of the kinase domain where many inhibitors establish hydrogen bonds to improve affinity and selectivity (Patel et al. 2017; Li et al. 2019). This moiety typically includes amine, amide, or other nitrogen‐containing groups interacting with the hinge region's residues, forming stable hydrogen bonds. The linker region provides flexibility and positions additional functional groups to reach different sub‐pockets in the EGFR binding site, enhancing binding stability (Shi et al. 2022; Karnik et al. 2021). It is often composed of carbon chains, ether, or other flexible bonds that allow the molecule to adapt to the binding pocket. This inhibitor region extends outward from the active site and interacts with surrounding residues or the solvent (Agarwal et al. 2022). Modifications in this area are crucial for enhancing selectivity, reducing off‐target effects, and overcoming resistance mutations. Common groups include halogen atoms (fluorine, chlorine) or polar groups interacting with solvent molecules. The structural components of EGFR inhibitors are primarily designed to optimize interactions with the ATP‐binding site while adding specificity and overcoming resistance through modifications in the hinge region, linker, and additional back‐pocket elements (Lu et al. 2020; Agarwal et al. 2022). Each generation of inhibitors builds on these fundamentals to improve efficacy and address emerging resistance in cancer treatment. The literature reports compound **1** with low IC_50_ values (0.278 μM for EGFR^WT^), emphasizing the critical role of the cyclohexyl group in achieving potent inhibition. This functional group enhances binding efficiency, stabilizes interactions with EGFR, and significantly contributes to the compound's high potency (Elmetwally et al. 2019; Mostafa et al. 2024). Similarly, compound **2** demonstrated the importance of the cyclohexyl group in ensuring high binding efficiency within the hydrophobic pocket. Its strategic position and functionality are pivotal for overcoming mutations, such as T790M, which alter the kinase domain's shape and chemical environment (Nasser et al. 2020). Adel and co‐researchers further validated this design strategy by investigating pyrimidine derivatives targeting EGFRᵀ^790^ᴹ. Notably, compound **3**, which incorporates a terminal cyclohexyl tail, exhibited remarkable potency against both wild‐type and mutant forms of EGFR, particularly EGFRᵀ^790^ᴹ. The hydrophobic channel formed by Phe723, Glu758, Ile759, and Glu762 was effectively occupied by the terminal cyclohexyl tail, underscoring its significance in molecular recognition and binding stability (Xu et al. 2020). Furthermore, compounds **4–8** (Figure 3) (Elmetwally et al. 2019; Mostafa et al. 2024; Othman et al. 2021; Zhang, Lv, et al. 2018), reported by various researchers, highlight the fundamental pharmacophores of EGFR inhibitors. These studies emphasize the critical roles of the NH spacer and hydrophobic tail in achieving effective binding affinity. Structural modifications in lead molecules have been shown to improve IC_50_ values, underlining the importance of fragment‐based optimization in rational drug design. This foundation provides a blueprint for developing related analogs that exploit hydrophobic regions within EGFR's binding pocket, enabling further advancements in targeted therapies.

The X‐ray crystal structures of the mutant EGFR T790M kinase domain bound to the third‐generation TKI inhibitors WZ4002 and Osimertinib revealed that the pyrimidine core adopts a distinctive U‐shaped conformation. This configuration includes an aniline ring bearing a hydrophilic group and an acrylamide moiety (Lu et al. 2020; Mansour et al. 2023). Among EGFR inhibitors, U‐shaped and Y‐shaped compounds refer to specific binding conformations or designs tailored to interact optimally with different forms of mutated or overactive EGFR (Mansour et al. 2023; Nitulescu et al. 2023). U‐shaped inhibitors are typically designed to bind in a compact U‐shaped conformation in the ATP‐binding site. They often bridge interactions between the hinge region and the back pocket. Erlotinib and Gefitinib are classic examples that fit in a U‐shape within the ATP‐binding pocket. These drugs are effective against certain EGFR mutations, especially in NSCLC (Nitulescu et al. 2023). Whereas Y‐shaped inhibitors are structurally distinct, occupying both the ATP‐binding site and an adjacent allosteric pocket. The shape allows the inhibitor to anchor at the hinge while extending into additional sites within the EGFR. Osimertinib is a notable example of a Y‐shaped inhibitor designed to overcome resistance caused by mutations like T790M. Therefore, utilizing these strategies, we have designed our target molecules (Milik et al. 2017; Nitulescu et al. 2023; Kardile et al. 2023).

Therefore, to rationalize the EGFR inhibitor's potency, we have designed the titled compounds by utilizing both strategies. The central aromatic heterocycle (highlighted in pink) is a core structural element across standard compounds like gefitinib, erlotinib, and afatinib, ensuring binding affinity to the kinase active site (Pawara et al. 2022; Islam 2020). Specific substituents such as halogens (Cl, F) and amino groups contribute to interaction with residues in the active site, affecting both potency and selectivity. They provide a balance of hydrophobic and polar interactions. The design of **Ri–Rxii** involves fragment‐based optimization, seen in modifications on either the hydrophobic head (cyan), central core, or hydrophobic tail regions. The selection of fragments should enhance specific interactions with mutant or resistant forms of EGFR, as illustrated by structural variations in compounds like rociletinib and osimertinib. The NH spacer (highlighted in the bottom section) is critical for maintaining an appropriate spatial arrangement, allowing for flexibility in the binding and positioning of the functional groups (Li et al. 2020). This central hetero‐aromatic system acts as a scaffold for diversification. Modifications that counter specific mutations (e.g., T790M in EGFR) are evident in compounds like Osimertinib (Zhang et al. 2012). For **Ri–Rxii**, designing modifications that incorporate structural rigidity or polar groups could enhance selectivity against resistant mutants, an increasingly common issue in targeted therapy. Moreover, both WZ4002 and Osimertinib share a U‐shaped core that provides an ideal fit for the ATP‐binding pocket of the EGFR kinase. This U‐shaped design is key for achieving potent and selective binding by maximizing interactions with essential residues within the active site (Nitulescu et al. 2023; Patil et al. 2024). For **Ri–Rxii**, maintaining this U‐shaped core is critical. It serves as the central framework around which functional groups can be appended to enhance binding affinity and selectivity for EGFR. The structure‐based approach leverages spatial orientation, hydrophobicity, and specific mutation‐targeting elements to achieve an optimized EGFR inhibitor.

## 2 Results and Discussion

### 2.1 Chemistry

**SCHEME 1:**
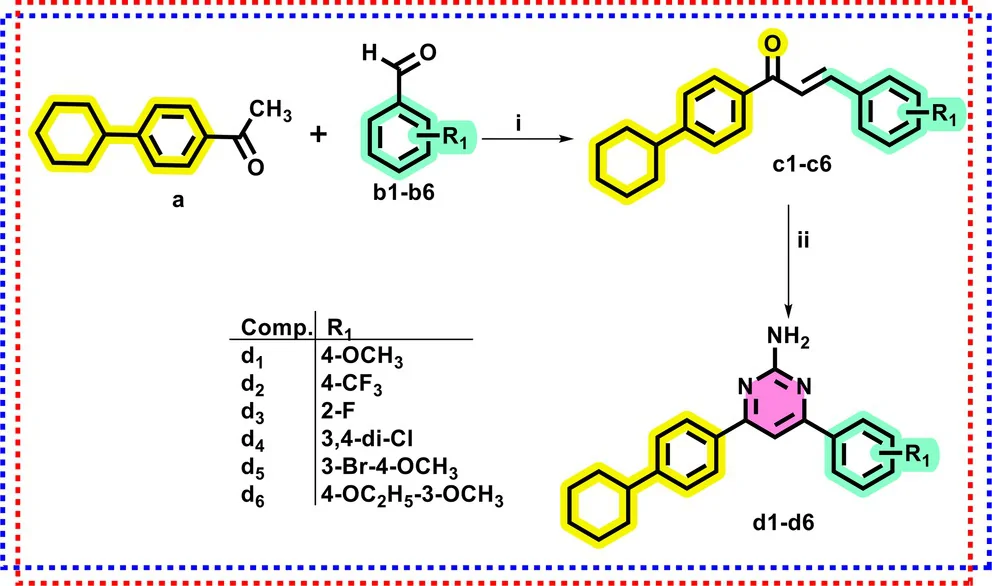
Synthetic route for the synthesis of 4‐(4‐substituted phenyl)‐6‐(4‐cyclohexylphenyl)pyrimidin‐2‐amine (**d1**–**d6**). Reagents and conditions: (i) ethanol, 30% NaOH, 0°C–5°C, 4–5 h; (ii) guanidine hydrochloride, ethanol, 30% NaOH, reflux, 10–12 h.

The synthesis of 4‐(4‐substituted phenyl)‐6‐(4‐cyclohexylphenyl)pyrimidin‐2‐amine*N*‐Cinnamamide/benzamide‐(4‐(substituted phenyl)‐6‐(4‐cyclohexylphenyl)pyrimidin‐2‐yl) (**d1–d6**) is represented in Scheme 1 and compounds were achieved through a two‐step process. First, chalcones (**c1–c6**) were synthesized via Claisen‐Schmidt condensation between 4‐cyclohexylacetophenone (**a**) and various substituted benzaldehydes (**b1–b6**) in ethanol using 30% NaOH at 0°C–5°C for 4–5 h. The resulting chalcones were then cyclized with guanidine hydrochloride in ethanol under reflux conditions in the presence of 30% NaOH for 10–12 h, yielding the desired pyrimidin‐2‐amine derivatives.

**SCHEME 2:**
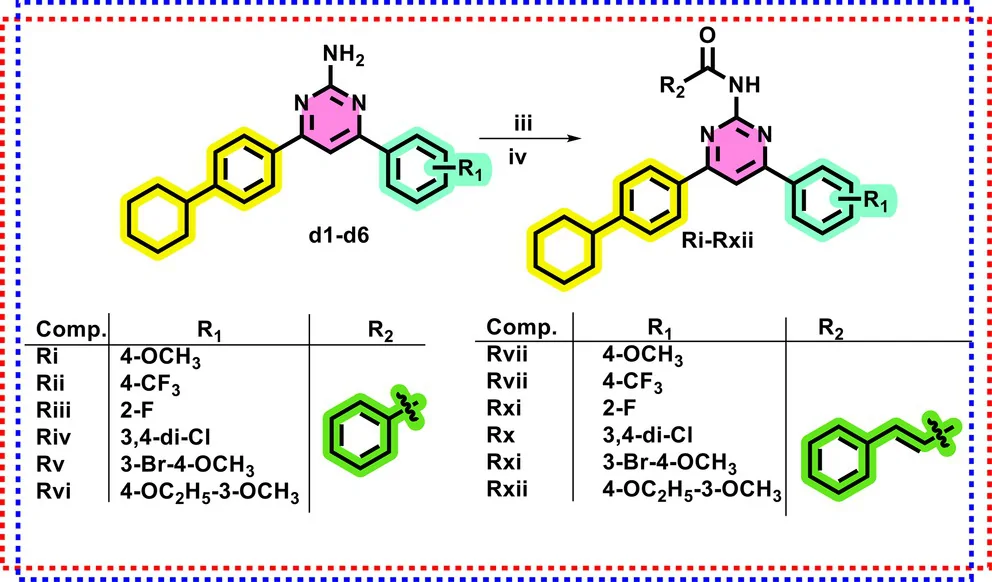
Synthetic route of the target compounds (**Ri**–**Rxii**). Reagents and conditions: (iii) Benzoyl chloride/Cinnamoyl chloride, DMF, K_2_CO_3_, 90°C–100°C, 14–26 h, (iv) 0°C–5°C, dilute HCl, stirring, 15–30 min.

Further, the synthesis of the target compounds **Ri–Rxii** was performed through the reaction from intermediates **d1–d6** with benzoyl chloride or cinnamoyl chloride in the presence of DMF as a solvent and potassium carbonate (K_2_CO_3_) as a base at 90°C–100°C for 14–26 h (Scheme 2). This resulted in the formation of N‐benzoyl or N‐cinnamoyl derivatives. In the next step, the reaction mixture was cooled to 0°C–5°C, followed by treatment with dilute HCl under stirring for 15–30 min, leading to the precipitation of the target compounds **Ri–Rxii**. The novel derivatives were identified using FTIR, ^1^H NMR, ^13^C NMR, and HRESI‐MS. The physiochemical parameter of final derivatives (**Ri–Rxii**) is represented in Table 1.

**TABLE 1:** Physicochemical properties of *N*‐Cinnamamide/benzamide‐(4‐(substituted phenyl)‐6‐(4‐cyclohexylphenyl)pyrimidin‐2‐yl) derivatives (**Ri–Rxii**).

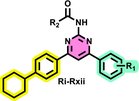								
Comp.	R_1_	R_2_	Molecular formula	Mol. wt.^a^	Color	Melting point (°C)	*R*_f_ (EA: PE)	% Yield
**Ri**	4‐OCH_3_	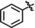	C_30_H_29_N_3_O_2_	463.58	Yellow	298–300	0.37	65
**Rii**	4‐CF_3_		C_30_H_26_F_3_N_3_O	501.55	White	250–252	0.39	69
**Riii**	2‐F		C_29_H_26_FN_3_O	451.55	Orange	269–271	0.31	62
**Riv**	3,4‐di‐Cl		C_29_H_25_Cl_2_N_3_O	502.44	White	268–270	0.25	63
**Rv**	3‐Br‐4‐OCH_3_		C_30_H_28_BrN_3_O_2_	542.48	Brown	272–274	0.29	60
**Rvi**	4‐OC_2_H_5_–3‐OCH_3_		C_32_H_33_N_3_O_3_	507.63	Dark brown	245–247	0.35	66
**Rvii**	4‐OCH_3_	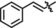	C_32_H_31_N_3_O_2_	489.62	Dark Yellow	276–278	0.31	72
**Rviii**	4‐CF_3_		C_32_H_28_F_3_N_3_O	527.59	White	266–268	0.30	75
**Rix**	2‐F		C_31_H_28_FN_3_O	477.58	Reddish brown	286–288	0.38	78
**Rx**	3,4‐di‐Cl		C_31_H_27_Cl_2_N_3_O	528.48	White	262–624	0.37	70
**Rxi**	3‐Br‐4‐OCH_3_		C_32_H_30_BrN_3_O_2_	568.52	Brown	268–371	0.35	78
**Rxii**	4‐OC_2_H_5_‐3‐OCH_3_		C_34_H_35_N_3_O_3_	533.67	Yellow	300–302	0.27	79

Abbreviations: EA, ethyl acetate; PE, petroleum ether; *R*_f_, relative flow.

^a^ Molecular weight.

#### 2.1.1 Antiproliferative Activity

The antiproliferative activity of the synthesized compounds **Ri**–**Rxii** was assessed against a panel of cancer cell lines with varying EGFR mutations, A549 (EGFR^WT^), NCI‐H1975 (EGFR^L858R/T790M^), HCC827 (EGFR^Del E746‐A750^), and NCI‐H1975 (EGFR^L858R/T790M/C797S^) (Table 2). These mutations represent EGFR activation, resistance to first/s‐generation inhibitors, and resistance to third‐generation inhibitors (EGFR^C797S^). Compound **Rix** (IC_50_: 3.48 ± 06.74 μM) and **Riv** (IC_50_: 8.99 ± 03.25 μM) exhibited the strongest antiproliferative activity among all compounds against wild‐type EGFR, suggesting these compounds may efficiently target native receptor conformations. While, compounds **Riii**, **Rx**, and **Rii** demonstrated moderate activity with IC_50_ values of 9.25 **±** 63.75, 13.25 ± 90.04, and 12.03 ± 78.56 μM respectively indicating some affinity for wild‐type EGFR. Further, compounds **Rix** (IC_50_: 3.67 ± 23.18 μM), **Rx** (IC_50_: 4.24 ± 27.76 μM), **Riv** (IC_50_: 9.89 ± 86.36 μM), and **Riii** (IC_50_: 10.88 **±** 95.25 μM) exhibited the strongest activity against NCI‐H1975 cell lines suggesting, these compounds likely stabilize interactions with the mutant receptor, overcoming steric hindrance induced by the T790M “gatekeeper” mutation. Compounds **Riii** (IC_50_: 0.17 **±** 11.36 μM) and **Riv** (IC_50_: 1.01 **±** 14.36 μM) demonstrated outstanding potency against EGFR^Del E746‐A750^, a deletion mutation commonly associated with sensitivity to EGFR inhibitors. Whereas, **Rix** (IC_50_: 2.54 ± 36.36 μM), **Rx** (IC_50_: 1.08 ± 42.36 μM), and **Rii** (IC_50_: 1.55 ± 16.65 μM) also exhibited strong activity, reflecting their compatibility with the altered active site caused by the deletion mutation. Similarly, compounds **Rix** (IC_50_: 2.24 **±** 0.62 μM), **Rx** (IC_50_: 2.75 **±** 0.12 μM), **Riv** (IC_50_: 2.76 ± 8.44 μM), and **Riii** (IC_50_: 2.83 ± 6.24 μM) showed a strong affinity towards triple‐mutant EGFR variant, which is resistant to third‐generation inhibitors. Their potency highlights the potential of these compounds as next‐generation EGFR inhibitors. Compounds **Rii** (IC_50_: 4.18 ± 2.31 μM), **Ri** (IC_50_: 8.89 ± 08.34 μM), and **Rviii** (IC_50_: 9.55 ± 0.85 μM) also demonstrated moderate effectiveness against the NCI‐H1975 cell line. Overall, compounds containing 2‐fluro (**Rix** and **Riii**), and 3,4‐di chloro (**Riv** and **Rx**) displayed promising antiproliferative activity against various EGFR‐mutant cell lines, including third‐generation resistance mutations (C797S).

**TABLE 2:** Antiproliferative activity of *N*‐Cinnamamide/benzamide‐(4‐(substituted phenyl)‐6‐(4‐cyclohexylphenyl)pyrimidin‐2‐yl) derivatives (**Ri–Rxii**) against NSCLC cell lines.

Comp	Antiproliferative activity IC_50_ ± SEM (μM)^a^			
	A549 (EGFR^WT^)	NCI‐H1975 (EGFR^L858R/T790M^)	HCC827 (EGFR Del E746‐A750)	NCI‐H1975 (EGFR^L858R/T790M/C797S^)
**Ri**	15.99 ± 78.21	15.95 ± 21.36	02.80 ± 08.34	08.89 ± 08.34
**Rii**	12.03 ± 78.56	15.65 ± 58.36	01.55 ± 16.65	4.18 ± 2.31
**Riii**	09.25 ± 63.75	10.88 ± 95.25	00.17 ± 11.36	2.83 ± 6.24
**Riv**	08.99 ± 03.25	09.89 ± 86.36	01.01 ± 14.36	2.76 ± 8.44
**Rv**	16.75 ± 75.25	16.22 ± 63.96	03.18 ± 16.36	NT^b^
**Rvi**	28.24 ± 98.51	27.89 ± 86.02	08.50 ± 49.35	NT^b^
**Rvii**	30.78 ± 21.03	35.33 ± 86.36	07.09 ± 98.36	NT^b^
**Rviii**	14.85 ± 36.04	18.74 ± 25.30	03.08 ± 68.85	9.55 ± 0.85
**Rix**	3.48 ± 06.74	3.67 ± 23.18	02.54 ± 36.36	2.24 ± 0.62
**Rx**	13.25 ± 90.04	4.24 ± 27.76	01.08 ± 42.36	2.75 ± 0.12
**Rxi**	46.25 ± 36.84	44.56 ± 25.25	09.87 ± 25.36	NT^b^
**Rxii**	41.58 ± 36.04	42.22 ± 03.58	09.99 ± 08.45	NT^b^
Gefitinib	1.79 ± 00.35	9.18 ± 01.06	NT^b^	NT^b^
Afatinib	NT^b^	2.05 ± 01.89	1.53 ± 01.05	NT^b^
Osimertinib	00.59 ± 02.34	0.09 ± 03.58	0.001 ± 0.10	2.02 ± 0.10

^a^ IC_50_ values are the mean ± SD of three separate experiments.

^b^ Compounds not tested for their antiproliferative activities.

#### 2.1.2 In Vitro Cytotoxicity Assay Against Human Normal Cell Lines

Compounds **Ri**, **Rii**, **Riii**, **Riv**, **Rv**, **Rviii**, **Rix**, and **Rx** were screened against the human mammary gland epithelium cell line (MCF‐10A), human kidney epithelial (T293) cell line, and the normal lung (WI‐38 and BEAS‐2B) cell lines (Table 3). Compounds **Riii**, **Riv**, **Rix**, and **Rx** showed safety among all the screened derivatives against all the cell lines. Compounds **Riii**, **Riv**, and **Rx** are selective for cancer cell lines over normal cells, with strong antiproliferative activity in sensitive EGFR mutant cancer cells (HCC827 and NCI‐H1975) and minimal toxicity in normal cells (Table S1 and Figure S1).

**TABLE 3:** Cytotoxicity assay of potent anticancer derivatives against human mammary gland epithelium cell line (MCF‐10A), human kidney epithelial (T293) cell line, and the normal lung (WI‐38 and BEAS‐2B) cell lines.

Compounds	IC_50_ (μM)^a^			
	Human mammary gland epithelium cell line	Human kidney epithelial cell line	Human lung cell line	
	MCF‐10A	T293	BEAS‐2B	WI‐38
**Ri**	89.23 ± 20.21	76.32 ± 10.11	55.68 ± 00.95	52.32 ± 00.89
**Rii**	86.57 ± 00.29	56.45 ± 08.31	59.78 ± 01.00	57.36 ± 01.56
**Riii**	90.24 ± 08.01	96.12 ± 08.01	90.75 ± 02.01	100.56 ± 00.86
**Riv**	89.56 ± 10.11	85.36 ± 08.23	92.21 ± 01.38	100.02 ± 02.32
**Rv**	72.36 ± 11.21	60.58 ± 11.20	45.56 ± 00.89	49.36 ± 01.56
**Rviii**	90.99 ± 01.21	85.96 ± 10.31	49.36 ± 0.99	52.24 ± 01.25
**Rix**	90.21 ± 08.28	87.32 ± 03.91	89.63 ± 02.58	94.36 ± 2.36
**Rx**	87.45 ± 07.20	85.99 ± 04.01	88.36 ± 01.25	96.58 ± 03.25
Erlotinib	78.02 ± 06.11	76.25 ± 01.21	79.21 ± 00.85	78.02 ± 00.28
Osimertinib	98.01 ± 01.36	99.00 ± 01.10	> 100	> 100

^a^ IC_50_ values are the mean ± SD of three separate experiments.

#### 2.1.3 Enzyme Inhibition Activity

The presented data highlights the enzyme inhibition activity (IC_50_ values) of four compounds (**Riii**, **Riv**, **Rix**, and **Rx**) against different EGFR mutations, including **EGFR** ^**WT**^, **EGFR** ^**T790M**^, **EGFR** ^**L858R/T790M**^, and **EGFR** ^**L858R/T790M/C797S**^ (Table 4). These clinically significant mutations represent various states of EGFR‐driven cancer progression and acquired resistance to EGFR inhibitors. Compound **Riii** demonstrated moderate inhibition of wild‐type EGFR (IC_50_: 01.99 ± 0.01 μM) and slightly reduced potency against the resistant mutation **EGFR** ^**T790M**^ (IC_50_: 2.02 **±** 02.01 μM). Compound **Riii** demonstrated the highest potency against the EGFR^L858R/T790M^ mutant with an IC_50_ value of 0.079 ± 0.01 μM. However, its inhibitory activity was markedly reduced against the EGFR^L858R/T790M/C797S^ triple mutant, exhibiting an IC_50_ of 8.98 ± 0.01 μM. This represents approximately a 114‐fold decrease in potency toward the triple mutant compared to EGFR^L858R/T790M^, indicating strong selectivity for the double mutant form. This profile showed strong activity against early EGFR mutations, but was less effective against the C797S mutation, which confers resistance to third‐generation EGFR inhibitors. Compound **Riv** emerged as the most potent inhibitor in the series. It displayed an IC_50_ of 0.56 ± 0.11 μM against EGFR^WT^ and 01.01 ± 0.03 μM against EGFR^T790M^, indicating moderate activity toward both forms. Remarkably, **Riv** demonstrated substantially enhanced potency against the EGFR^L858R/T790M^ mutant, with an IC_50_ of 0.030 ± 0.003 μM, corresponding to an approximately 19‐fold improvement over EGFR^WT^ and a 33‐fold improvement over EGFR^T790M^. Importantly, **Riv** retained considerable inhibitory activity against the EGFR^L858R/T790M/C797S^ triple mutant, exhibiting an IC_50_ of 1.07 ± 0.02 μM. This reflects only a ~1.9‐fold decrease in potency relative to EGFR^WT^ and a modest ~36‐fold reduction compared to the double mutant, representing the smallest activity loss among the tested compounds. This suggests **Riv** could serve as a lead candidate for targeting both primary EGFR mutations and resistant triple mutants. Conversely, compounds **Rix** and **Rx** exhibited high IC_50_ values and poor selectivity (ratios of 5.91 and 5.99, respectively), reflecting their limited potential for overcoming resistance. Among the reference drugs, Osimertinib achieves remarkable selectivity with an IC_50_ of 0.77 ± 00.12 μM for EGFR^L858R/T790M/C797S^. This highlights that compound **Riv** has low IC_50_ values and favorable selectivity, making it a strong candidate for further development as a next‐generation EGFR inhibitor.

**TABLE 4:** In vitro enzymatic inhibitory activities of **Riii**, **Riv**, **Rix**, and **Rx** against EGFR^WT^, EGFR^T790M^, EGFR^L858R/T790M^, and EGFR^L858R/T790M/C797S^.

Comp	In vitro enzyme inhibition activity IC_50_ (μM)^a^			
	EGFR^WT^	EGFR^T790M^	EGFR^L858R/T790M^	EGFR^L858R/T790M/C797S^
**Riii**	01.09 ± 0.01	02.02 ± 02.01	0.079 ± 01.01	8.98 ± 01.26
**Riv**	0.56 ± 01.11	01.01 ± 0.03	0.030 ± 0.003	01.07 ± 00.02
**Rix**	10.21 ± 01.01	17.06 ± 03.91	20.33 ± 01.38	60.36 ± 2.66
**Rx**	09.95 ± 01.02	10.90 ± 01.01	22.05 ± 01.20	59.58 ± 01.05
Erlotinib	00.55 ± 01.02	00.96 ± 0.289	NT^b^	NT^b^
Gefitinib	NT^b^	18.01 ± 0.78	NT^b^	NT^b^
Afatinib	00.18 ± 1.25	01.21 ± 1.56	NT^b^	NT^b^
Osimertinib	NT^b^	NT^b^	0.0011 ± 0.01	0.77 **±** 00.12

^a^ Data were expressed as mean ± standard error (SE) of three independent experiments.

^b^ Compounds not tested for their enzyme inhibition activity.

#### 2.1.4 Cell Cycle Analysis

**FIGURE 4:**
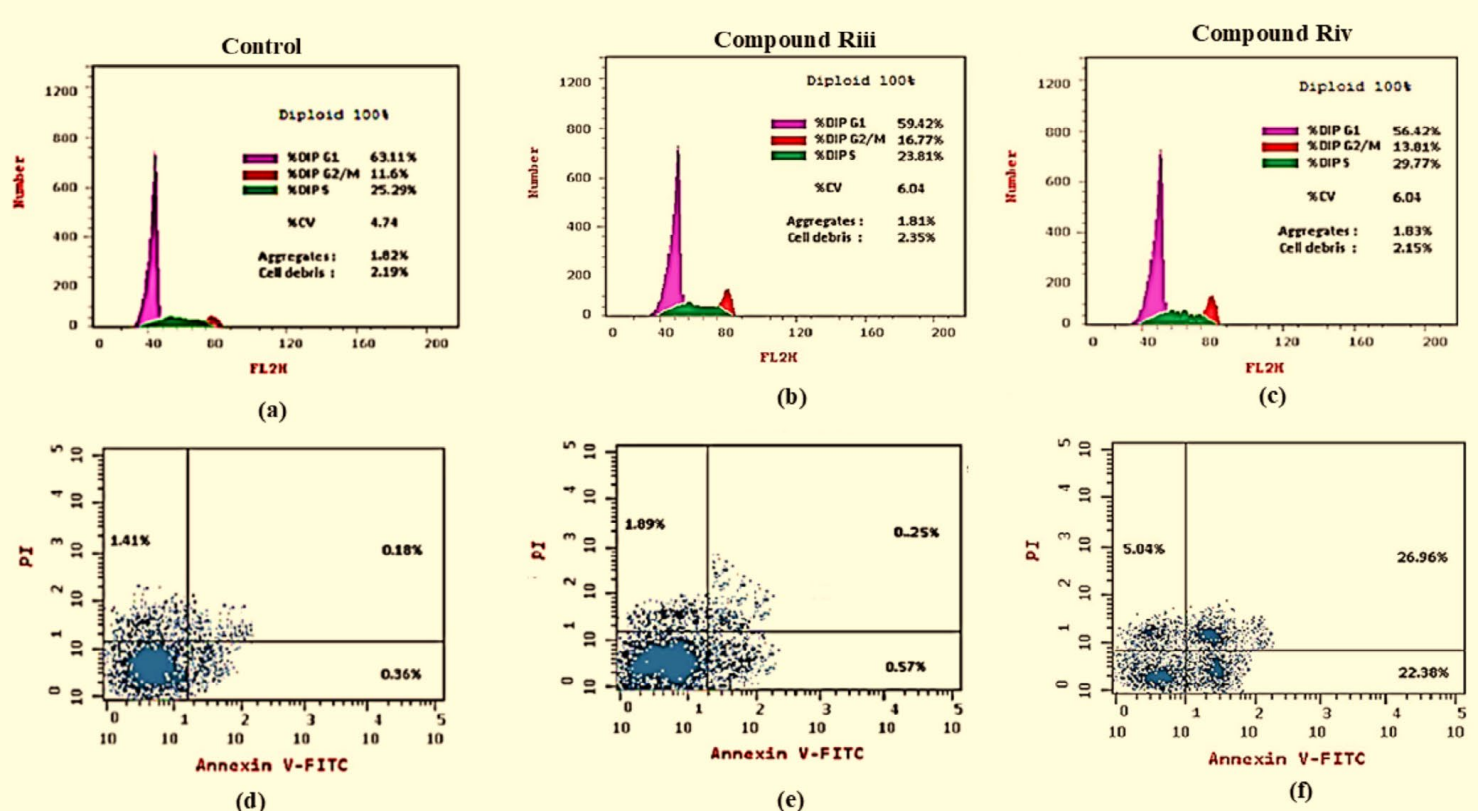
(a–c) Cell cycle analysis showing G1, S, and G2/M phase distribution for control, compound **Riii**, and compound **Riv**, respectively. (d–f) Apoptosis analysis using Annexin V‐FITC/PI staining demonstrates live, early apoptotic, late apoptotic, and necrotic cell populations for control, compound **Riii**, and compound **Riv**, respectively.

**FIGURE 5:**
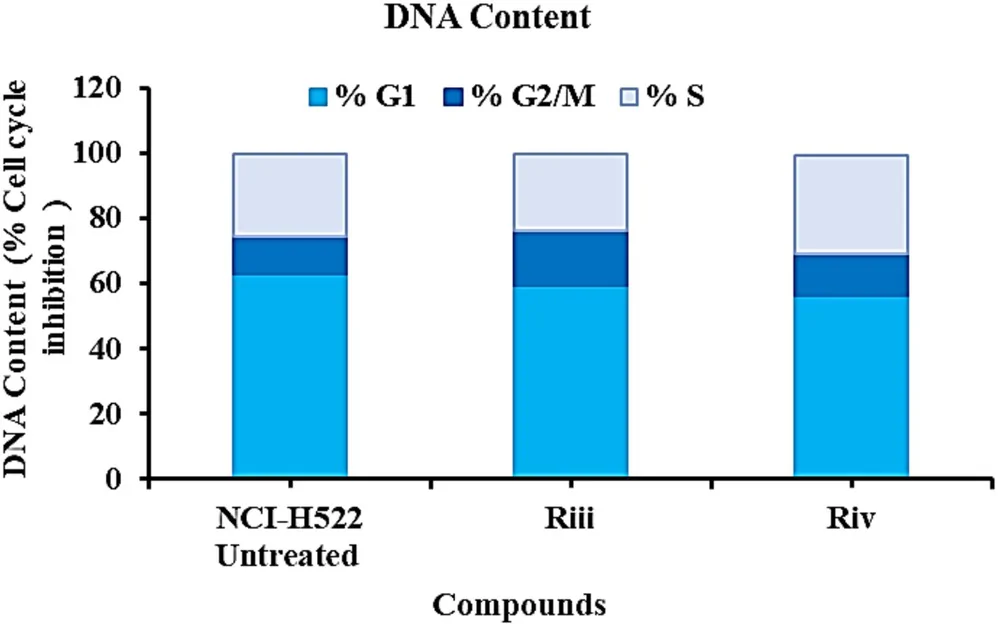
Effect of compounds (**Riii** and **Riv**) on cell cycle progression in NCI‐H1975 cells, measured as the percentage of cells in different phases of the cell cycle (% G1, % S, and % G2/M). Cells were treated with 10 μM of each compound for the indicated 24 h. Data indicate cell‐cycle inhibition relative to untreated control cells.

The cell cycle analysis revealed that both compounds **Riii** and **Riv** effectively induced cell cycle arrest in NCI‐H1975 cells, albeit at different phases (Figure 4). In the untreated control group, the majority of cells were in the G1 phase (63.11%), with a smaller proportion in the G2/M (11.6%) and S phases (25.29%). Treatment with **Riii** resulted in a reduction in G1 phase cells (59.42%) and an increase in the G2/M phase population (16.77%), indicating that **Riii** induces cell growth arrest primarily at the G2/M phase. On the other hand, **Riv** caused a more substantial shift in the cell cycle, with a reduction in the G1 phase population (56.42%) and an increase in both the S phase (29.77%) and G2/M phase (13.01%), suggesting that **Riv** induces arrest in both the S and G2/M phases (Figure 5). These findings indicate that **Riv** has a broader impact on disrupting cell cycle progression compared to compound **Riii** (Table 5).

**TABLE 5:** Cell cycle inhibition results of compound **R12**.

Compound	DNA Content			
	% G1	% G2/M	% S	Comment
Cont. NCI‐H1975 (untreated)	63.11	11.6	25.29	—
**Riii**	59.42	16.77	23.81	Cell growth arrest at the G2/M phase
**Riv**	56.42	13.01	29.77	Cell growth arrest at the G2/M and S phase
Osimertinib	Osimertinib exposure at IC_50_ (3 μM) for 72 h increased G0/G1 phase in A549 cells^a^			

^a^ Cited from Nanamiya et al. (2021).

#### 2.1.5 Apoptosis Assay

**FIGURE 6:**
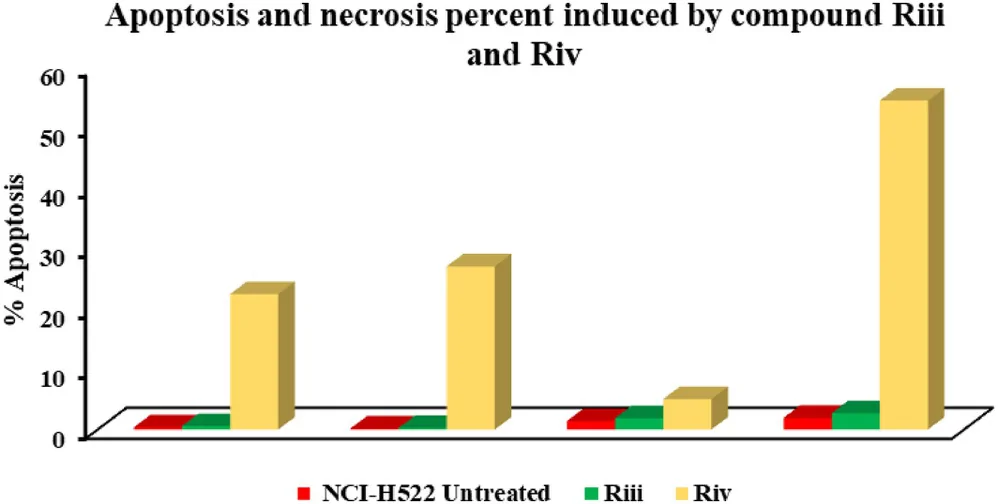
Induction of apoptosis and necrosis in NCI‐H1975 cells by compounds **Riii** and **Riv**. The graph compares the percentage of apoptotic cells in untreated samples versus those treated with **Riii** and **Riv**, highlighting the significant effect of **Riv** in inducing apoptosis. Cells were treated with 10 μM of each compound for the indicated 24 h.

The apoptosis analysis further demonstrated **Riv**'s superior pro‐apoptotic activity. In the untreated control group, the total percentage of cells undergoing apoptosis or necrosis was minimal (1.93%). Treatment with **Riii** slightly increased the total apoptotic and necrotic population to 2.71%, reflecting its limited ability to induce cell death. Conversely, **Riv** significantly elevated both early apoptosis (22.38%) and late apoptosis (26.96%), with a total apoptotic and necrotic population of 54.38%, including 5.04% necrosis (Figure 6). This indicated that **Riv** induced robust apoptotic cell death, far exceeding the effects observed with **Riii** (Figure 6). Taken together, these results highlight the dual activity of **Riv**, inducing both cell cycle arrest (at the S and G2/M phases) and apoptosis, making it a promising candidate for further anticancer evaluation (Table 6).

**TABLE 6:** Apoptosis and necrosis percentages induced by compounds **Riii** and **Riv**.

Compound	Apoptosis %^a^			
	Early	Late	Necrosis	Total
Cont. NCI‐H1975 (untreated)	0.36	0.16	1.41	1.93
**Riii**	0.57	0.25	1.89	2.71
**Riv**	22.38	26.96	5.04	54.38
Osimertinib	Osimertinib treatment (5 μM) increased apoptosis in NCI‐H1975 cells from ~5.1% to ~11.2% as measured by flow cytometry (Annexin V/PI)^b^			

^a^ Values are given as the mean of three different experiments.

^b^ Cited from Sun et al. (2020).

### 2.2 Antioxidant Activity

**FIGURE 7:**
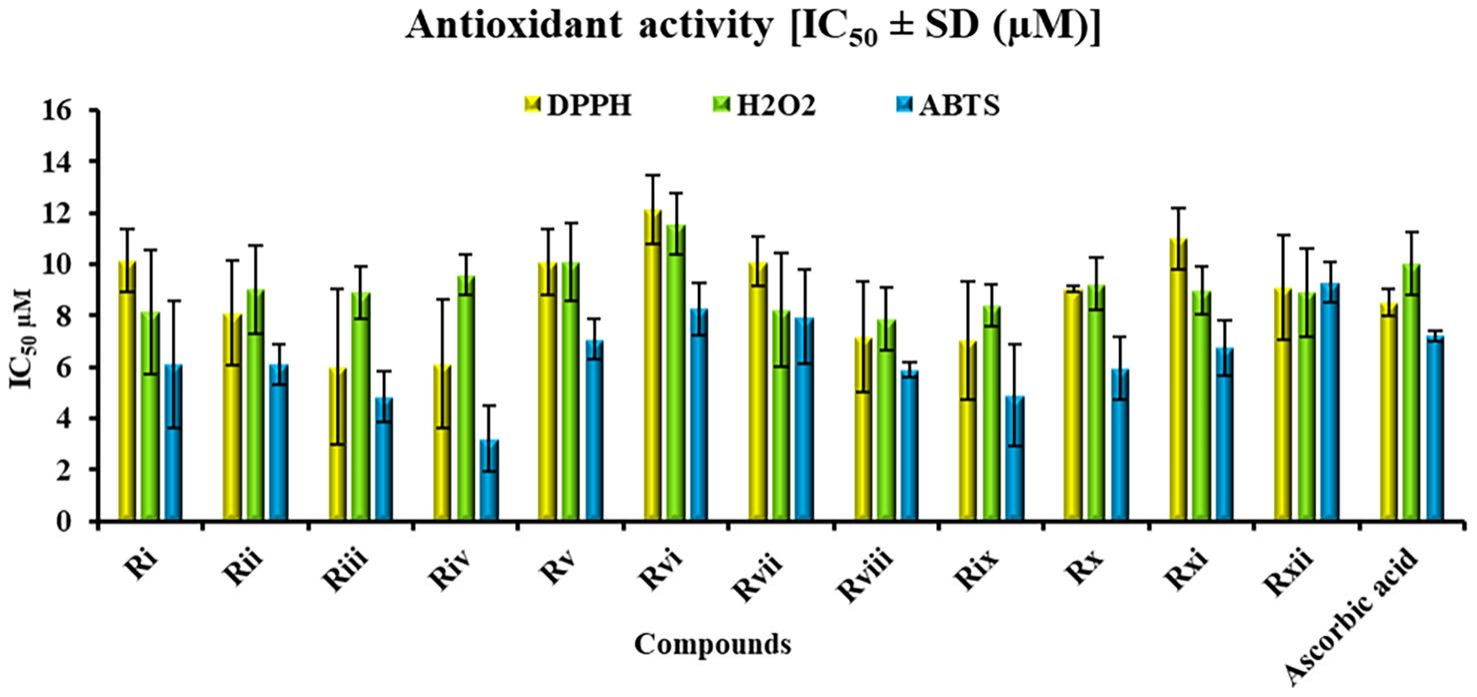
Antioxidant activity of various compounds (**Ri** to **Rxii**) and ascorbic acid evaluated by three assays (DPPH, H_2_O_2_, and ABTS), expressed as IC_50_ values (μM) with standard deviation (SD).

Antioxidant assays like DPPH, H_2_O_2_, and ABTS measure the ability of compounds to scavenge free radicals or reduce oxidative stress. Their relevance to cancer lies in the critical role that reactive oxygen species (ROS) play in cancer development, progression, and treatment. Elevated ROS levels in cells can cause DNA damage, mutations, and oxidative stress, all of which contribute to carcinogenesis. While cancer cells often rely on a balance of ROS for growth and survival, disrupting this balance through antioxidant or pro‐oxidant mechanisms can influence cancer cell fate. The DPPH assay assesses free radical scavenging ability, the H_2_O_2_ assay measures the reduction of hydrogen peroxide, a contributor to oxidative DNA damage, and the ABTS assay reflects the compound's electron‐donating capacity and inhibition of lipid peroxidation. Together, these assays provide insight into compounds' potential to modulate oxidative stress, which may complement other anticancer mechanisms, such as EGFR inhibition. Furthermore, by neutralizing reactive oxygen species (ROS), antioxidants prevent DNA damage, lipid peroxidation, and protein oxidation, reducing the risk of mutations and cancer initiation. They also protect normal cells from oxidative damage caused by chemotherapy or radiation therapy, thereby improving treatment safety. Additionally, antioxidants can selectively induce cancer cell death by pushing ROS levels beyond a lethal threshold. Therefore, we have analyzed the antioxidant activity of our compounds by three different methods, and most of the titled derivatives represent higher antioxidant potency (Table 7). Across all assays, several compounds demonstrated significant antioxidant activity compared to the standard, ascorbic acid (Figure 7).

**TABLE 7:** Determination of antioxidant activity through DPPH, H_2_O_2_, and ABTS assay for compounds **Ri–RXii**.

Compounds	Antioxidant activity IC_50_ ± SD (μM)^a^		
	DPPH	H_2_O_2_	ABTS
**Ri**	10.15 ± 1.22	08.15 ± 2.42	06.10 ± 2.47
**Rii**	08.10 ± 2.06	09.01 ± 1.73	06.11 ± 0.78
**Riii**	06.00 ± 3.05	08.89 ± 1.02	04.82 ± 0.99
**Riv**	06.11 ± 2.52	09.57 ± 0.78	03.21 ± 1.27
**Rv**	10.09 ± 1.30	10.06 ± 1.52	07.08 ± 0.77
**Rvi**	12.13 ± 1.32	11.56 ± 1.21	08.26 ± 1.02
**Rvii**	10.11 ± 0.95	08.22 ± 2.22	07.95 ± 1.82
**Rviii**	07.17 ± 2.16	07.89 ± 1.23	05.90 ± 0.29
**Rix**	07.04 ± 2.29	08.38 ± 0.82	04.89 ± 1.98
**Rx**	09.01 ± 0.12	09.21 ± 1.02	05.95 ± 1.21
**Rxi**	11.00 ± 1.19	08.96 ± 0.92	06.75 ± 1.08
**Rxii**	09.10 ± 2.06	08.89 ± 1.72	09.28 ± 0.79
Ascorbic acid	08.52 ± 0.01	10.02 ± 1.20	07.21 ± 0.22

^a^ Data were expressed as IC_50_ ± standard error (SE) of three independent experiments.

For DPPH radical scavenging, the most potent compounds were **Riii** (6.00 ± 3.05 μM), **Riv** (6.11 ± 2.52 μM), and **Rix** (7.04 ± 2.29 μM), outperforming ascorbic acid (8.52 ± 0.01 μM). Compounds **Ri**, **Rv**, **Rvii**, and **Rx** showed moderate activity, with IC₅₀ values ranging from 9.01 to 10.15 μM, while **Rvi** and **Rxi** were the least effective, with IC₅₀ values above 11 μM. In the H_2_O_2_ scavenging assay, most compounds exhibited activity comparable to or better than ascorbic acid (10.02 ± 1.20 μM). Compounds **Ri** (8.15 ± 2.42 μM), **Riii** (8.89 ± 1.02 μM), **Rix** (8.38 ± 0.82 μM), and **Rviii** (7.89 ± 1.23 μM) demonstrated strong activity. However, **Riv** and **Rii** showed slightly weaker activity, with IC_50_ values of 9.57 ± 0.78 μM and 9.01 ± 1.73 μM, respectively. Compound **Rvi** was the least active compound in this assay (11.56 ± 1.21 μM). For the ABTS radical scavenging assay, **Riv** (3.21 ± 1.27 μM) was the most potent compound, exhibiting substantially stronger activity than ascorbic acid (7.21 ± 0.22 μM) and all other compounds. **Riii** (4.82 ± 0.99 μM) and **Rix** (4.89 ± 1.98 μM) also showed notable potency. Other compounds, including **Rv**, **Rvii**, and **Rxi**, had moderate activity with IC₅₀ values ranging from 5.90 to 7.95 μM. **Rxii** was the least effective in this assay (9.28 ± 0.79 μM).

Overall, **Riv** exhibited the strongest and most consistent antioxidant activity across all assays, particularly excelling in the ABTS assay. **Riii** and **Rix** also showed strong antioxidant potential in multiple assays.

### 2.3 Nitric Oxide Release Assay

The percentage of nitric oxide (NO) release by the synthesized compounds (**Ri–Rxii**) was evaluated using the Griess assay over a time‐dependent interval of 5–50 min, and the results are expressed as mean ± SD. As shown in Table 8, all tested compounds exhibited a time‐dependent increase in NO release, indicating a gradual and sustained generation of nitric oxide with increasing incubation time.

**TABLE 8:** Percentage of NO release of compounds (**Ri–Rxii**).

Compound	(%) Percentage of NO release of compounds ± SD^a^									
	Time (min)									
	5	10	15	20	25	30	35	40	45	50
**Ri**	12 ± 2.1	22 ± 2.8	31 ± 3.2	38 ± 3.5	45 ± 3.8	51 ± 4.0	56 ± 4.1	60 ± 4.2	64 ± 4.2	67 ± 4.3
**Rii**	15 ± 2.0	26 ± 2.7	35 ± 3.1	42 ± 3.4	48 ± 3.7	54 ± 3.9	59 ± 4.0	63 ± 4.1	66 ± 4.2	69 ± 4.2
**Riii**	28 ± 1.8	42 ± 2.4	53 ± 2.7	62 ± 2.9	69 ± 3.0	74 ± 3.1	78 ± 3.1	81 ± 3.2	84 ± 3.2	86 ± 3.2
**Riv**	30 ± 1.7	45 ± 2.3	56 ± 2.6	65 ± 2.8	72 ± 2.9	77 ± 3.0	81 ± 3.0	84 ± 3.1	87 ± 3.1	89 ± 3.1
**Rv**	11 ± 2.2	20 ± 2.9	28 ± 3.3	35 ± 3.6	41 ± 3.9	46 ± 4.1	50 ± 4.2	54 ± 4.3	57 ± 4.3	60 ± 4.4
**Rvi**	9 ± 2.3	17 ± 3.0	24 ± 3.4	30 ± 3.7	36 ± 4.0	41 ± 4.2	45 ± 4.3	48 ± 4.4	51 ± 4.4	53 ± 4.5
**Rvii**	13 ± 2.1	23 ± 2.8	32 ± 3.2	39 ± 3.5	46 ± 3.8	52 ± 4.0	57 ± 4.1	61 ± 4.2	65 ± 4.2	68 ± 4.3
**Rviii**	20 ± 1.9	32 ± 2.6	42 ± 3.0	50 ± 3.2	57 ± 3.4	62 ± 3.6	66 ± 3.7	70 ± 3.8	73 ± 3.8	75 ± 3.9
**Rix**	22 ± 1.9	34 ± 2.5	44 ± 2.9	52 ± 3.1	59 ± 3.3	64 ± 3.5	68 ± 3.6	71 ± 3.7	74 ± 3.7	76 ± 3.8
**Rx**	16 ± 2.0	28 ± 2.7	37 ± 3.1	44 ± 3.4	50 ± 3.7	56 ± 3.9	61 ± 4.0	65 ± 4.1	68 ± 4.2	70 ± 4.2
**Rxi**	14 ± 2.1	25 ± 2.8	34 ± 3.2	41 ± 3.5	47 ± 3.8	53 ± 4.0	58 ± 4.1	62 ± 4.2	66 ± 4.2	69 ± 4.3
**Rxii**	12 ± 2.1	21 ± 2.8	30 ± 3.2	37 ± 3.5	44 ± 3.8	50 ± 4.0	55 ± 4.1	59 ± 4.2	63 ± 4.2	66 ± 4.3
Ascorbic acid	18 ± 1.9	30 ± 2.6	40 ± 3.0	48 ± 3.2	55 ± 3.4	60 ± 3.6	65 ± 3.7	69 ± 3.8	72 ± 3.8	74 ± 3.9

^a^ % of nitric oxide release ± standard error (SE) of three independent experiments.

NO is a well‐recognized biological mediator that plays a dual role in cancer biology, where sustained and elevated NO levels are associated with antiproliferative, pro‐apoptotic, and anti‐angiogenic effects. In the present study, the NO‐releasing ability of the synthesized compounds showed a clear time‐dependent increase, which can be directly correlated with their observed anticancer potential. Among the tested compounds, **Riii** and **Riv** demonstrated the highest NO‐releasing capacity, showing a rapid onset and significantly higher NO release compared to other derivatives. Compound **Riv** exhibited the maximum NO release (89% ± 3.1%) at 50 min, followed closely by **Riii** (86% ± 3.2%), surpassing the standard antioxidant ascorbic acid (74% ± 3.9%) at the same time point. This enhanced activity suggests that these compounds possess strong NO‐donating potential, possibly due to favorable structural features that facilitate nitric oxide generation. Compounds **Rviii** and **Rix** also showed notable NO release, reaching 75% ± 3.9% and 76% ± 3.8%, respectively, at 50 min, comparable to or slightly higher than ascorbic acid. In contrast, compounds **Rvi** and **Rv** exhibited relatively lower NO release throughout the experiment, indicating weaker NO‐donating ability. The superior performance of compounds **Riii** and **Riv** highlights them as promising NO‐releasing agents, which may contribute to their potential anticancer activity.

### 2.4 In Silico EGFR^WT^ , EGFR^T790M^ ^/L858R^, and EGFR^L858R^ ^/T790M/C797S^ Inhibition Properties

The in silico evaluation of the inhibition properties of compounds against EGFR^WT^, EGFR^T790M/L858R^, and EGFR^L858R/T790M/C797S^ revealed that compounds showed varying degrees of binding affinity and inhibitory activity across the different EGFR variants. In the case of EGFR^WT^ **(4I23)**, compound **Riv** exhibited the strongest binding affinity with a Δ*G* of −12.0 kcal/mol and a *Ki* of 1.56 nM, suggesting potent inhibition. Similarly, **Rii** and **Riii** also showed good binding, with Δ*G* values of −9.9 kcal/mol and −10.8 kcal/mol, respectively. For the EGFR^T790M/L858R^ (3W2O) variant, **Rix** demonstrated moderate binding (Δ*G* = −8.7 kcal/mol, *Ki* = 348.65 nM), while **Riv** again showed favorable binding with Δ*G* = −7.5 kcal/mol and *Ki* = 3135.86 nM, though not as strong as in EGFRWT. In the case of EGFR^L858R/T790M/C797S^ (6LUD), **Riv** remained the most promising compound with a Δ*G* of −10.4 kcal/mol and *Ki* of 23.35 nM, showing its potential to effectively inhibit resistant EGFR mutants. Other compounds like **Rix** and **Rii** displayed moderate to strong activity, supporting their role as potential candidates for overcoming resistance mechanisms, including the challenging C797S mutation. Overall, these results indicate that certain compounds, particularly **Riv**, demonstrated significant inhibition against both wild‐type and resistant EGFR variants, highlighting their potential as effective inhibitors for targeted cancer therapies (Table 9).

**TABLE 9:** In silico EGFR^WT^, EGFR^T790M/L858R^, and EGFR^L858R/T790M/C797S^ blocking properties of compounds **Ri–Rxii**.

Compounds	4I23 (EGFR^WT^)		3W2O (EGFR^T790M/L858R^)		6LUD (EGFR^L858R/T790M/C797S^)	
	Δ*G* (kcal mol^−1^)	*K*_*i*_ (nM)	Δ*G* (kcal mol^−1^)	*K*_*i*_ (nM)	Δ*G* (kcal mol^−1^)	*K*_*i*_ (nM)
**Ri**	−9.6	90.2271	−7.8	1888.9103	−10.0	45.8996
**Rii**	−9.9	54.3489	−9.8	348.6521	−10.3	27.6479
**Riii**	−10.8	11.8782	−8.0	248.6729	−10.3	27.6479
**Riv**	−12.0	01.5637	−7.5	3135.8628	−10.4	23.3497
**Rv**	−9.7	76.2000	−7.9	1595.2525	−10.1	38.7639
**Rvi**	−9.2	177.3636	−7.0	7299.0543	−9.5	106.8363
**Rvii**	−9.9	54.3489	−7.0	7299.0543	−9.6	90.2271
**Rviii**	−10.4	23.3497	−7.6	2648.3486	−10.3	27.6479
**Rix**	−10.5	19.7196	−8.7	348.6521	−10.2	32.7375
**Rx**	−10.6	16.6539	−8.8	488.8280	−10.1	38.7639
**Rxi**	−10.2	32.7375	−8.6	488.8280	−9.9	54.3489
**Rxii**	−9.8	64.3536	−9.0	248.6729	−9.5	106.8363
Gefitinib	−9.4	126.5030	−8.5	578.8126	−7.4	3713.1198
Afatinib	−9.0	248.6729	−8.9	294.4492	−7.6	2648.3486
Osimertinib	−8.2	960.9122	−8.6	488.8280	−7.8	1888.9103

### 2.5 Structure–Activity Relationship (SAR)

**FIGURE 8:**
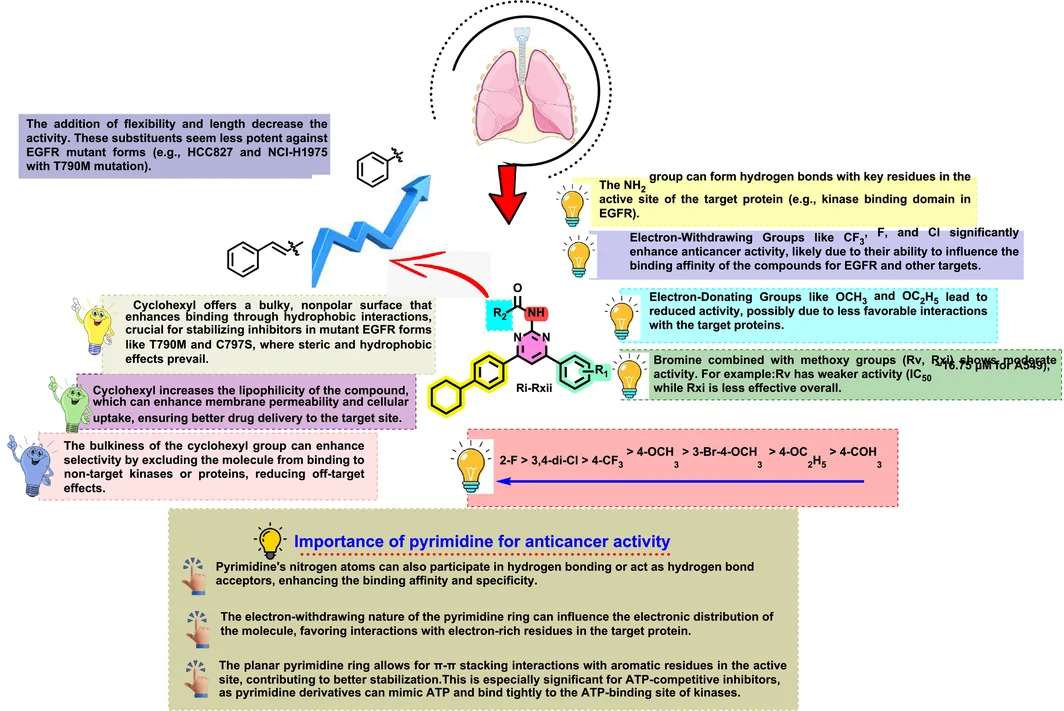
Structural insights and functional role of key substituents (pyrimidine, R_1_, R_2_, and cyclohexyl groups) of the target compounds *N*‐Cinnamamide/benzamide‐(4‐(substituted phenyl)‐6‐(4‐cyclohexylphenyl)pyrimidin‐2‐yl) (**Ri–Rxii**) in enhancing anticancer activity as EGFR inhibitors.

Compound **Riii** and **Riv** consistently show potent antiproliferative activity against multiple EGFR mutant cell lines, with the lowest IC_50_ values, especially against sensitive and resistant EGFR mutations (e.g., EGFR^Del E746‐A750^ and EGFR^L858R/T790M^). Substituents like 2‐F (**Riii**) and 3,4‐di‐Cl (**Riv**) enhanced activity, suggesting the importance of electron‐withdrawing groups on the phenyl ring. Bulky groups (e.g., 4‐OC_2_H_5_–3‐OCH_3_ in **Rvi** and **Rxii**) decreased activity, likely due to steric hindrance affecting binding to the EGFR pocket. In enzyme inhibition activity also compounds **Riv** (3,4‐di‐Cl) and **Riii** (2‐F) also exhibited low IC_50_ values for EGFR^WT^ and mutant variants, indicating strong EGFR binding affinity. The increased activity of **Riii** and **Riv** is likely attributed to their substituents' electron‐withdrawing properties, which may stabilize interactions with EGFR active sites. Compounds with bulkier or lipophilic substituents (e.g., **Rvi** and **Rxii**) showed weaker inhibition, suggesting limited fit into the binding pocket. Further, in antioxidant activity, compounds **Riv** and **Riii** showed strong antioxidant activity in DPPH and ABTS assays, which may be related to their electron‐withdrawing substituents enabling radical stabilization. Substituents like 3‐Br‐4‐OCH_3_ and bulky alkoxy groups in **Rv** and **Rxi** lead to moderate or reduced activity. the overall trends in the title compounds suggest that electron‐withdrawing groups (e.g., F, Cl, CF_3_), enhanced both EGFR inhibitory and antioxidant activities. Smaller groups (e.g., F in **Riii**) were more effective than bulky ones (e.g., CF_3_ in **Rii** and **Rviii**). While electron‐donating groups (e.g., OCH_3_, OC_2_H_5_) showed moderate activity. Bulky donating groups (e.g., 4‐OC_2_H_5_–3‐OCH_3_ in **Rvi**) reduced potency due to steric hindrance. Moreover, **s**ubstituents like F and Cl provide the best combination of potency and physicochemical properties (Figure 8).

### 2.6 In Silico Prediction of Physiochemical Properties and ADMET

**FIGURE 9:**
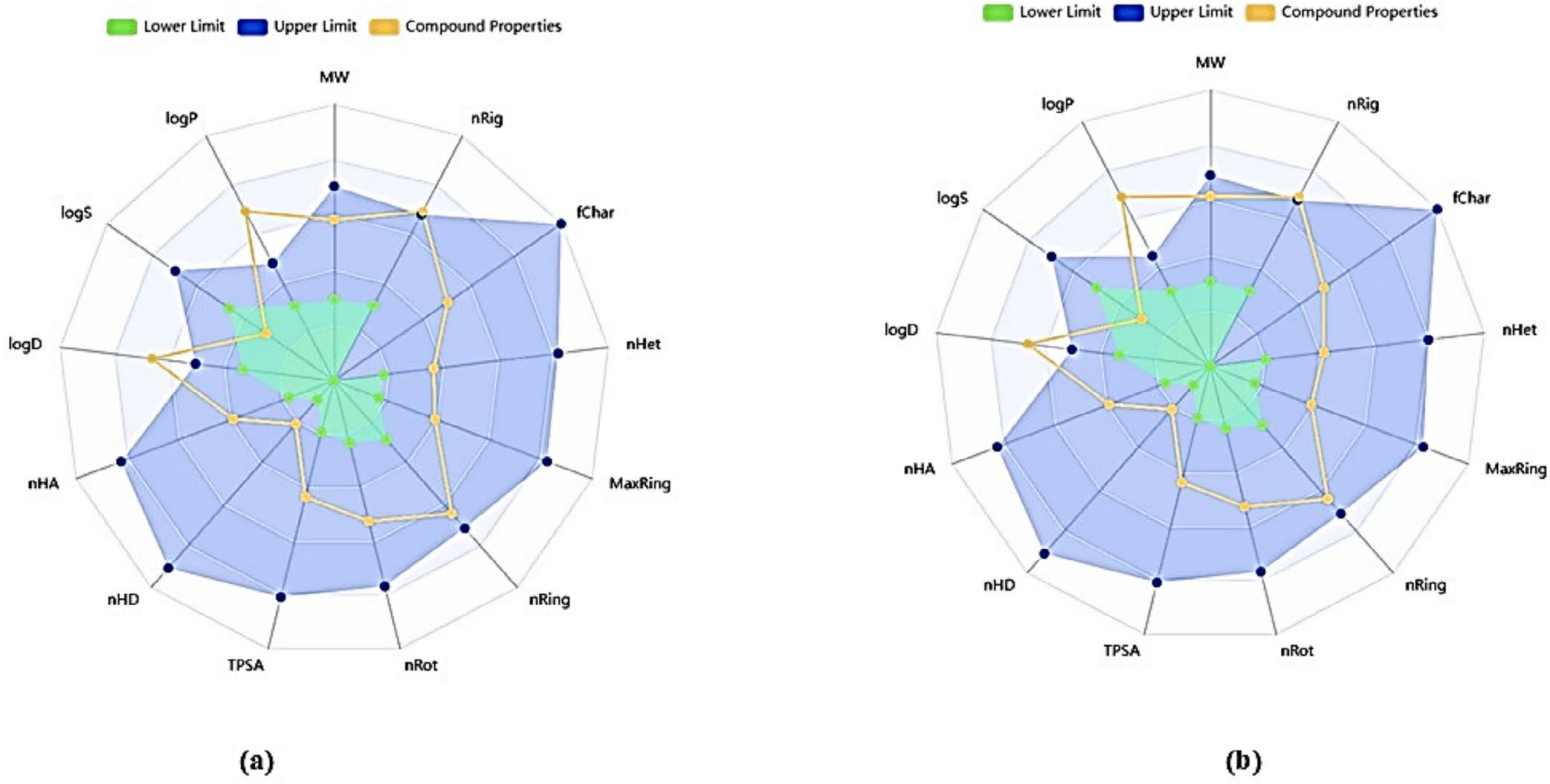
Radar plots representing the physicochemical properties of (a) compound **Riii** and (b) compound **Riv**. The green region indicates the lower limits, the blue region represents the upper limits, and the yellow plot illustrates the compound properties.

The ADMET profiles of the compounds **Riii** and **Riv** offer crucial insights into their pharmacokinetic properties and potential therapeutic application in anticancer therapy (Table 10). The ADMET profiles of **Riii** and **Riv** reveal important pharmacokinetic and safety considerations for their potential use in anticancer therapies. Both compounds have similar physicochemical properties. However, **Riv** is more lipophilic (higher logP), potentially enhancing its tissue penetration, though this may also limit solubility (Figure 9). Both compounds show low gastrointestinal absorption, with **Riii** slightly more soluble than **Riv**. They are Pgp substrates, suggesting they may be effluxed from cells, reducing bioavailability. Distribution studies show both compounds exhibit high plasma protein binding, with **Riv** having nearly 100% binding, potentially limiting free drug availability. **Riv** also demonstrates a higher volume of distribution, which may lead to broader tissue distribution.

**TABLE 10:** Physicochemical parameters of synthesized compounds (**Ri–Rxii**).

S. No.	Properties	Compounds	
		Riii	Riv
**1**.	**Physicochemical Property**		
a.	Molecular Weight (MW)	451.21	501.14
b.	Num. H‐bond acceptors	4	4
c.	Num. H‐bond donors	1	1
d.	Molar Refractivity	133.96	144.03
e.	TPSA (Å^2^)	54.88	54.88
f.	Fraction Csp3	0.21	0.21
g.	logS	−7.093	−8.604
h.	logP	6.685	8.05
**2**.	**Absorption**		
a.	Caco‐2 Permeability	−4.849	−4.893
b.	GI absorption	Low	Low
c.	Pgp inhibitor	No	No
d.	Pgp substrate	Yes	Yes
**3**.	**Distribution**		
a.	PPB	99.6%	100.0%
b.	VDss	2.977	4.002
c.	BBB permeant	No	No
d.	Fu	0.3%	0.1%
**4**.	**Metabolism**		
a.	CYP1A2 inhibitor	No	No
b.	CYP2C19 inhibitor	Yes	Yes
c.	CYP2C9 inhibitor	No	No
d.	CYP2D6 inhibitor	Yes	Yes
e.	CYP3A4 inhibitor	No	No
**5**.	**Excretion**		
a.	CL_plasma_	4.242	3.934
b.	T1/2	0.527	0.587
**6**.	**Toxicity**		
a.	Hepatotoxicity (dili)	Active (0.51)	Inactive (0.51)
b.	Neurotoxicity (neuro)	Active (0.84)	Active (0.85)
c.	Nephrotoxicity (nephro)	Inactive (0.79)	Inactive (0.78)
d.	Respiratory toxicity (respi)	Active (0.71)	Active (0.69)
e.	Cardiotoxicity (cardio)	Inactive (0.87)	Inactive (0.88)
e.	Carcinogenicity	Inactive (0.62)	Inactive (0.63)
f.	Immunotoxicity (immune)	Inactive (0.91)	Inactive (0.96)
g.	Mutagenicity (mutagen)	Inactive (0.72)	Inactive (0.73)
h.	Cytotoxicity (cyto)	Inactive (0.70)	Inactive (0.70)
i.	Predicted LD50	2000 mg/kg	2000 mg/kg
j.	Predicted Toxicity Class	4	4

*Note:* (a) Molecular weight; (b) Number of rotatable bonds; (c) Num. H‐bond acceptors; (d) Number of H‐bond donors; (e) Molar Refractivity; (f) Topological polar surface area; (g) Lipinski violations (MW: ≤ 500, Mlog P: ≤ 4.15, HBA: ≤ 10, and HBD: ≤ 5); (h) Viber rule (Rotatable bonds: ≤ 10 and TPSA: ≤ 140).

**FIGURE 10:**
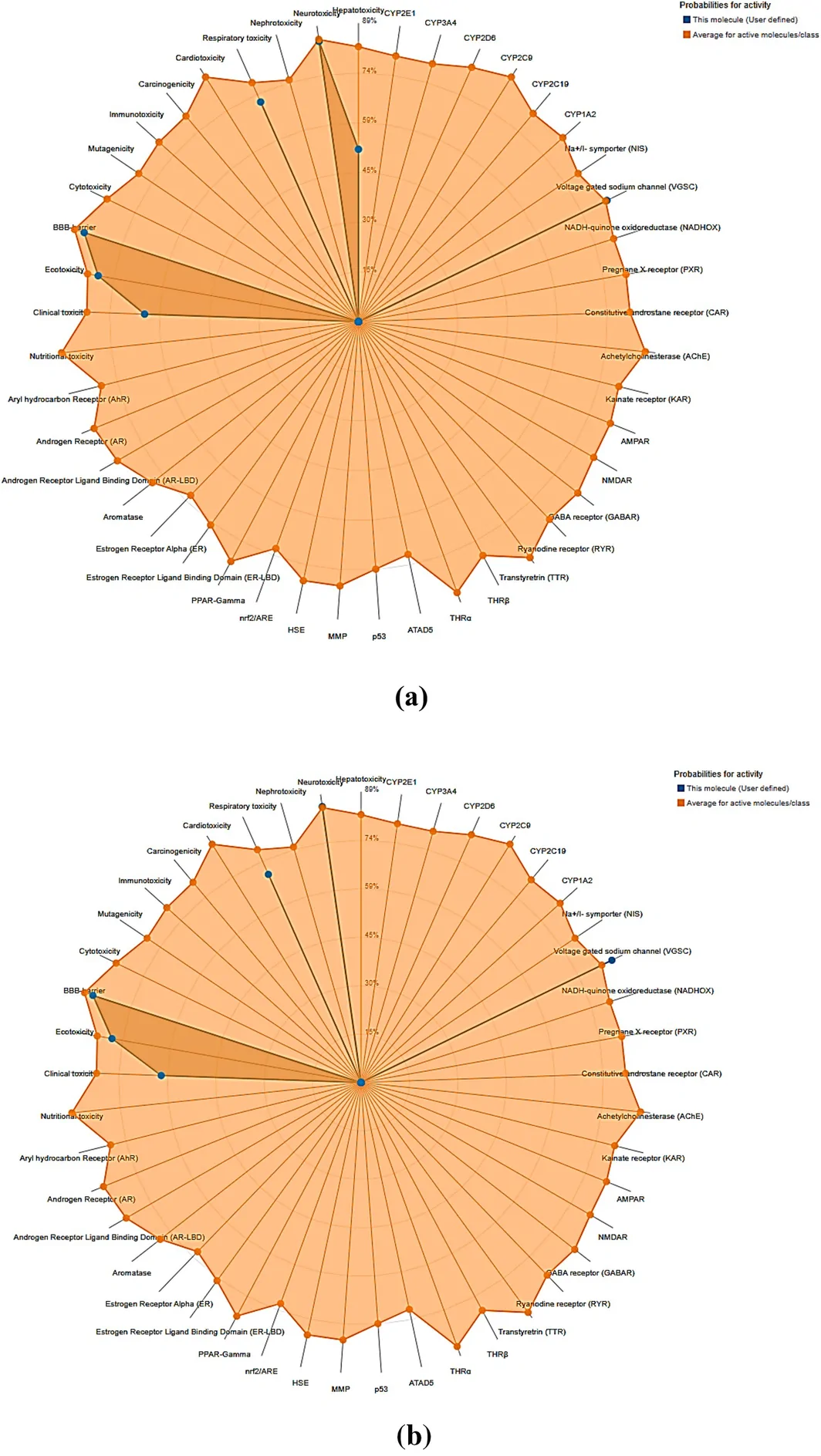
Radar plot illustrating the predicted toxicity profile of compound (a) **Riii**, and (b) **Riv**. The plot compares probabilities for activity (blue points) of the compound against average probabilities for active molecules in the respective classes (orange shaded region). Categories include toxicological endpoints, receptor binding, and enzymatic interactions.

Both compounds inhibit CYP2C19 and CYP2D6, which may lead to drug–drug interactions, but they do not affect other major metabolic enzymes. Their excretion profiles indicate rapid clearance with short half‐lives, supporting their swift elimination from the body. Toxicity analysis depicted in the radar for both compounds showed some neurotoxic and respiratory toxicity, but they are free from hepatotoxic, nephrotoxic, or cardiotoxic effects, suggesting a safer profile (Figure 10). Overall, **Riii** and **Riv** display promising pharmacokinetic properties for anticancer applications with manageable toxicity risks.

### 2.7 Docking Studies

The docking results reveal distinct binding affinities (ΔG) of the compounds against three EGFR variants: EGFR^WT^ (4I23), EGFR^T790M/L858R^ (3W2O), and EGFR^L858R/T790M/C797S^ (6LUD) (Table S1). Compound **Riv** exhibited the strongest binding affinity for EGFR^WT^ (−12.0 kcal/mol), outperforming all other compounds, suggesting its superior compatibility with the wild‐type receptor. Similarly, **Riii** and **Riv** demonstrated the most potent binding against the triple mutant EGFR^L858R/T790M/C797S^ (−10.3 and −10.4 kcal/mol, respectively), indicating their potential to overcome resistance associated with this variant. For the EGFR^T790M/L858R^ mutant, **Rii** displayed the best affinity (−9.8 kcal/mol), highlighting its efficacy against this clinically significant resistant mutation. In contrast, compounds such as **Rvi** and **Rvii** consistently showed weaker binding across all EGFR variants, with ΔG values ranging between −9.5 and −7.0 kcal/mol, suggesting limited therapeutic potential. Overall, **Riv** emerged as the most promising candidate with consistently high affinity across all variants, followed by **Riii** and **Rix**.

**FIGURE 11:**
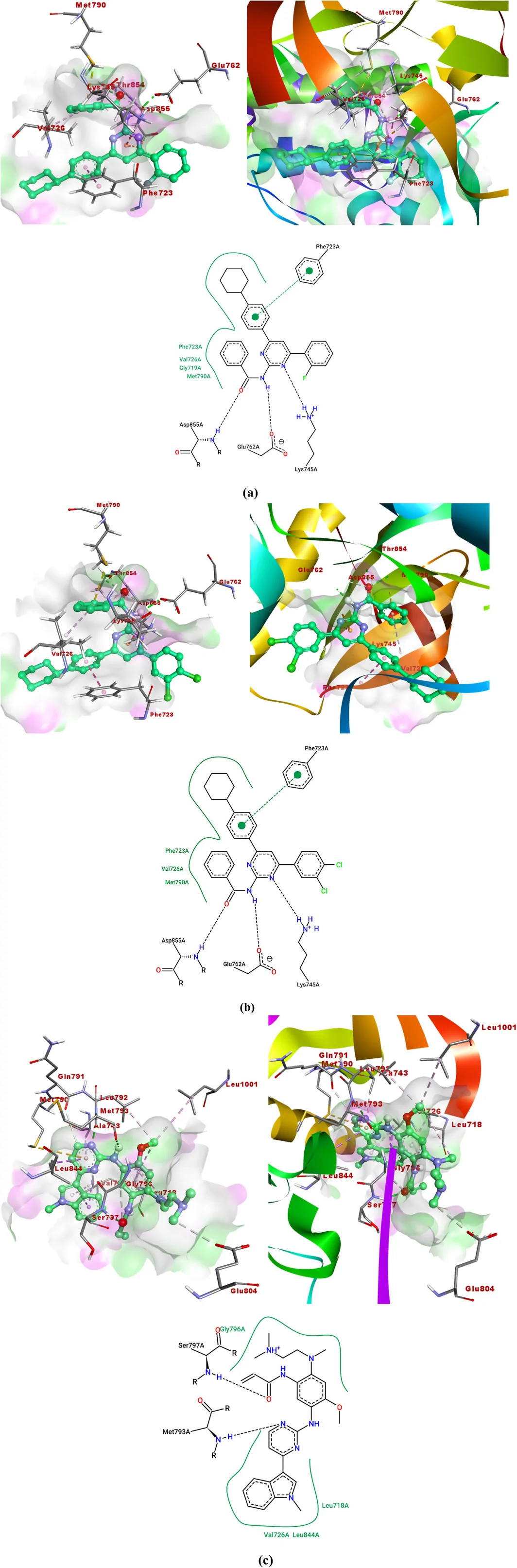
Molecular docking of compound (a) **Riii**, (b) **Riv**, and reference (c) Osimertinib.

The reference compound Osimertinib displayed a binding energy of −8.8 kcal/mol. Both compounds **Riii** and **Riv** demonstrated robust interactions through hydrogen bonding with key residues like Thr854 and Lys745, as well as pi‐pi stacking with Phe723, contributing to their higher affinities. In contrast, Osimertinib showed selective interactions, particularly with Thr790 and Met793, but fewer hydrophobic contacts, potentially accounting for its lower docking score (Figure 11). The additional interactions observed in **Riii** and **Riv** suggest enhanced binding potential, making them promising candidates for EGFR^L858R/T790M/C797S^ inhibitors. This highlights their potential to improve efficacy while maintaining or improving selectivity.

### 2.8 Molecular Dynamic Simulation Studies

The most promising compound, **Riv**, in complex with EGFR^L858R/T790M/C797S^, was subjected to molecular dynamics and normal mode simulations using the Desmond package from Schrödinger. Table 11 shows the average of several trajectories.

**TABLE 11:** Average of various MD simulation trajectories for **Riv** and EGFR^L858R/T790M/C797S^.

Complex	RMSD (Å)^a^		H‐bond^b^
	Protein (6LUD)	Ligand (Riv)	
**Riv**	2.21	4.62	2

^a^ Root mean square deviation.

^b^ Hydrogen bond.

#### 2.8.1 Root‐Mean‐Square Deviation (RMSD)

**FIGURE 12:**
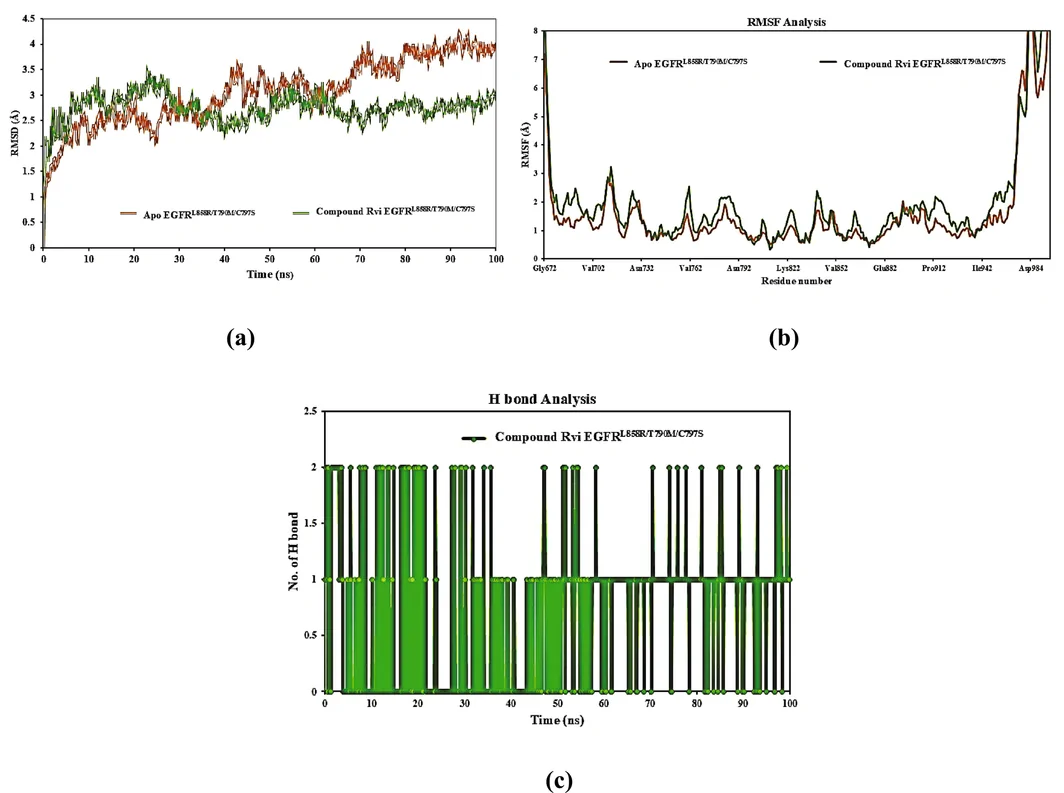
Molecular dynamics simulation of 100 ns of **Riv‐6LUD** complex. (a) RMSD of appo protein (PDB ID: 6LUD) and RMSD of Complex **Riv‐6LUD**; (b) RMSF of appo protein (PDB ID: 6LUD) and RMSD of Complex **Riv‐6LUD**; (c) H‐bonds.

The RMSD of the protein Cα atoms, derived from the simulated trajectory of the protein‐ligand complex, reflects the stability of the complex in a dynamic environment. Higher RMSD values for the protein Cα atoms suggest unfolding, while lower values indicate a more compact structure. The minimal fluctuation in the backbone RMSD supports the equilibration of the protein‐ligand complex. Backbone deviation is represented by the difference between the maximum and minimum RMSD values. The RMSD plot revealed a stable ligand‐protein complex throughout the simulation, with values ranging from 0.97 to 3.45 Å, and an average of 2.21 Å for the EGFR^L858R/T790M/C797S^ complex with **Riv**. In contrast, the RMSD values for the Apo protein Cα atoms of the EGFR^L858R/T790M/C797S^ complex ranged from 0.5 to 4.12 Å, with an average of 4.62 Å. The low RMSD value and minimal fluctuations indicate the complex's stability and consistency in a dynamic environment (Figure 12a).

#### 2.8.2 Root‐Mean‐Square Fluctuation (RMSF)

Individual amino acid residues within the protein‐ligand complex play a crucial role in maintaining the stability of dynamic processes. The RMSF values, calculated from the MD simulation trajectories, provide insight into the deviation of individual amino acids from their native structure. The RMSF plot is particularly useful for analyzing residual vibrations in the **Riv**‐protein complex.

Residues involved in rigid and well‐organized secondary structures, such as alpha helices and beta strands, exhibit minimal variation, as these regions are structurally stable. In contrast, loosely organized regions like loops, bends, and coils show higher RMSF values, indicating greater flexibility or instability. Despite minimal changes in the active site and main chain residues, the lead compound remained securely bound within the target protein's binding pocket, suggesting limited conformational changes and stable interaction. The RMSF plot highlights significant fluctuations in the C‐ and N‐terminal residues, as well as in loop regions, reflecting their inherent flexibility. In this regard, key residues interacting with **Riv** in EGFR binding sites, such as Met769, Leu820, Val702, Ala719, Ile765, Leu764, Cys773, Arg817, Asn818, Asp776, Asp863, Leu852, Lys753, Ala751, Cys805, and Val734 exhibited relatively rigid behavior, with low residue fluctuations (RMSF < 2.5 Å) throughout the MD simulation (Figure 12b). The minimal flexibility of these residues suggests that they maintained stable interactions with **Riv** throughout the MD simulation, in contrast to residues exhibiting greater fluctuations within the EGFR binding sites. The low RMSF values indicate that **Riv** remained appropriately accommodated within the protein‐binding pocket and maintained stable ligand–protein interactions during the simulation. These findings were consistent with the RMSD analysis, further supporting the overall stability of the **Riv–EGFR complexes**.

#### 2.8.3 Hydrogen Bonding Analysis

Hydrogen bonding plays a crucial role in determining the binding strength of a ligand to its target protein in a protein‐ligand complex. During the 100 ns MD simulation, compound **Riv** exhibited significant intermolecular hydrogen bonding with EGFR, ranging from 0 to 2 bonds. Figure 12c illustrates the number of hydrogen bonds and their distribution over the simulation timeframe. For the **Riv‐EGFR** ^**L858R/T790M/C797S**^ complex, the maximum and average number of hydrogen bonds were both observed to be 2. Hydrogen bond analysis not only supports the docking results but also confirms the structural stability of the binding sites, as no conformational changes were detected during the MD simulation.

### 2.9 DFT Analysis

The electronic properties and reactivity descriptors of the compound were analyzed using Density Functional Theory (DFT). The calculations were carried out with the B3LYP functional and the 6‐31G* basis set. The optimized geometry of the compound was used to determine the frontier molecular orbital (FMO) energies, such as the highest occupied molecular orbital (HOMO) and the lowest unoccupied molecular orbital (LUMO), along with other quantum chemical parameters.

**FIGURE 13:**
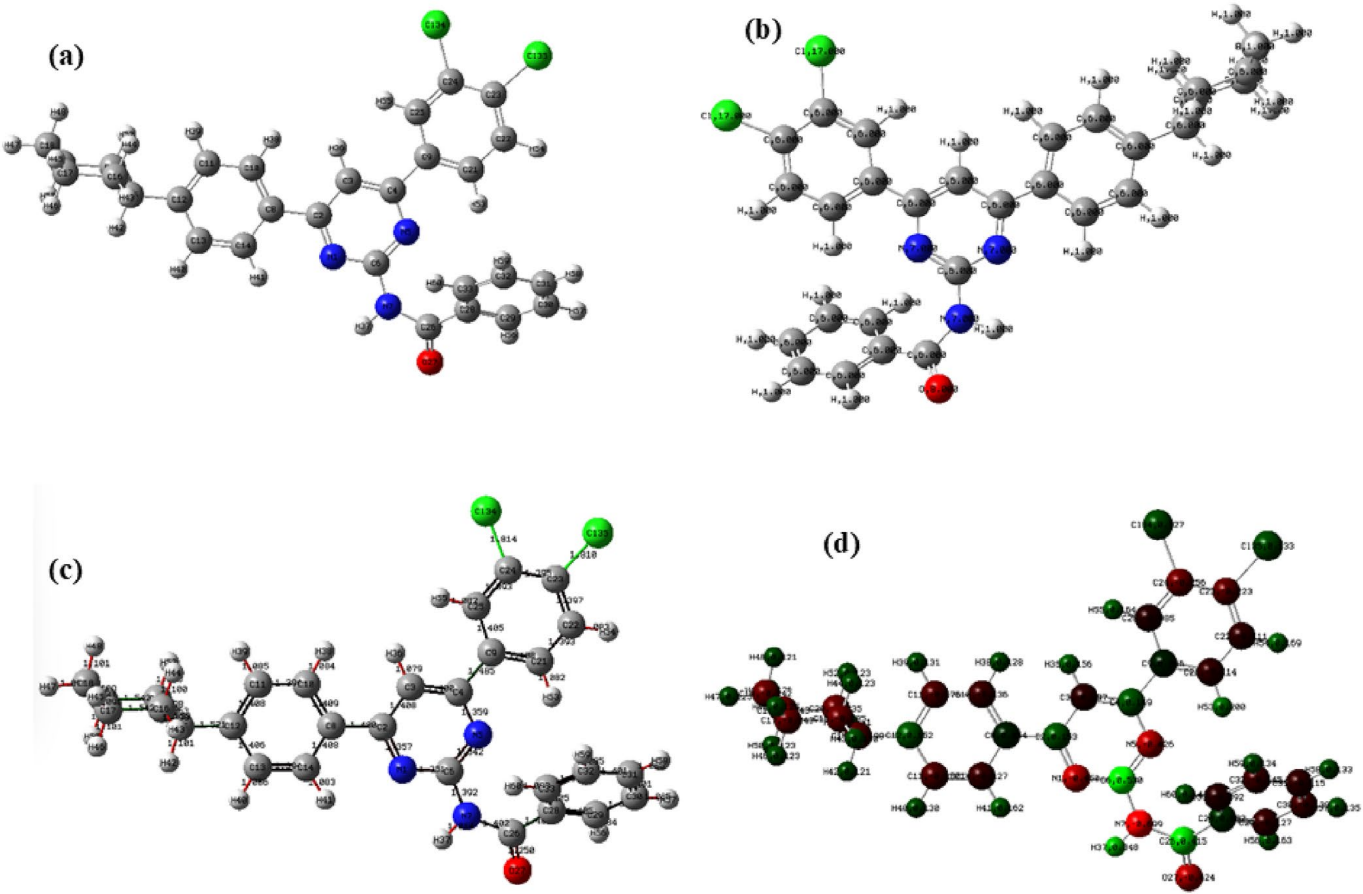
Optimized geometry of compound **Riv**. (a) Optimized molecular structure with atom labels, (b) Atom numbering for **Riv**, (c) Bond lengths between atoms in the structure, and (d) Atomic charges calculated using the Mulliken population analysis.

Figure 13 provides a detailed analysis of the optimized structure of the compound, offering insights into its atomic arrangement, bond lengths, and charge distribution, essential for understanding its potential biological activity. Figure 13a illustrates the optimized molecular structure with clear atom labels, where different elements such as carbon, nitrogen, oxygen, and hydrogen are identified. This geometry, optimized using DFT methods, is the foundation for further computational analyses. Figure 13b presents the atom numbering, which facilitates the precise identification of individual atoms for subsequent discussions on molecular interactions and reactivity. The numbering system is crucial for referencing specific atoms when analyzing their contributions to the molecule's overall properties. Figure 13c shows the bond lengths between atoms, providing valuable information on chemical bonds' strength and the molecule's structural stability. Shorter bond lengths indicate stronger interactions, essential for understanding the compound's behavior in biological systems. Figure 13d highlights the atomic charges calculated using Mulliken population analysis, revealing electron distribution across the molecule. This data is critical for identifying electron‐rich and electron‐deficient regions, offering insights into the molecule's potential for interactions with other molecules, such as receptors or enzymes. Together, these quantum chemical descriptors—optimized structure, bond lengths, and atomic charges—serve as essential tools in predicting the compound's reactivity, stability, and potential pharmacological activity, providing a comprehensive view of its molecular characteristics for drug design and development.

**FIGURE 14:**
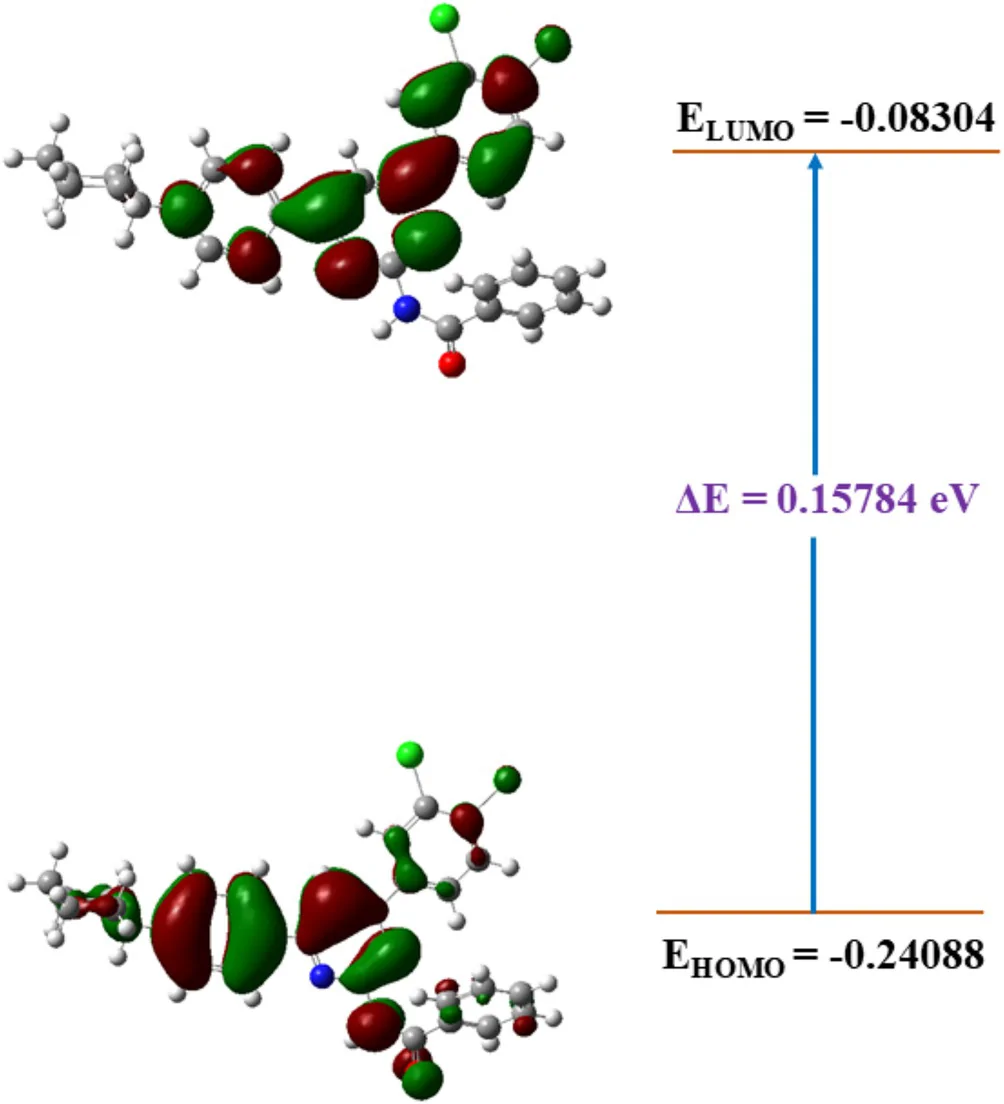
Frontier Molecular Orbitals of the Compound **Riv**. The top image represents the Lowest Unoccupied Molecular Orbital (LUMO) with an energy of −0.08304 eV, while the bottom image illustrates the Highest Occupied Molecular Orbital (HOMO) with an energy of −0.24088 eV. The energy gap (Δ*E*) between HOMO and LUMO is calculated as 0.15784 eV, indicating the compound's electronic excitation properties and chemical reactivity.

The obtained values are summarized in Table 12. The HOMO and LUMO energies were calculated to be −0.24088 eV and −0.08304 eV, respectively, resulting in a HOMO‐LUMO energy gap (Δ*E*) of 0.15784 eV0 (Figure 14). This small energy gap suggests moderate molecular stability and significant chemical reactivity, indicating the compound's potential for biological activity.

**TABLE 12:** DFT parameters for the **Riv**.

Parameter	HOMO (E_HOMO_)	LUMO (E_LUMO_)	Energy gap (ΔE)	Ionization potential (IP)	Electron affinity (EA)	Electronegativity (*χ*)	Chemical potential (*μ*)	Hardness (*η*)	Softness (S)	Electrophilicity index (*ω*)
Value (eV)	−0.24088	−0.083	0.15784	0.24088	0.08304	0.16196	−0.162	0.07892	12.67	0.1662

The ionization potential (IP) and electron affinity (EA) were calculated as 0.24088 and 0.08304 eV, respectively. These values reflect the compound's ability to donate and accept electrons. The electronegativity (*χ*) was found to be 0.16196 eV, indicating the compound's tendency to attract electrons. The chemical hardness (*η* = 0.07892 eV) and softness (*S* = 12.67 eV^−1^) were also calculated, demonstrating the molecule's polarizability and reactivity. Furthermore, the electrophilicity index (*ω* = 0.1662 eV) indicates the molecule's electrophilic nature, supporting its potential as a biologically active agent.

**FIGURE 15:**
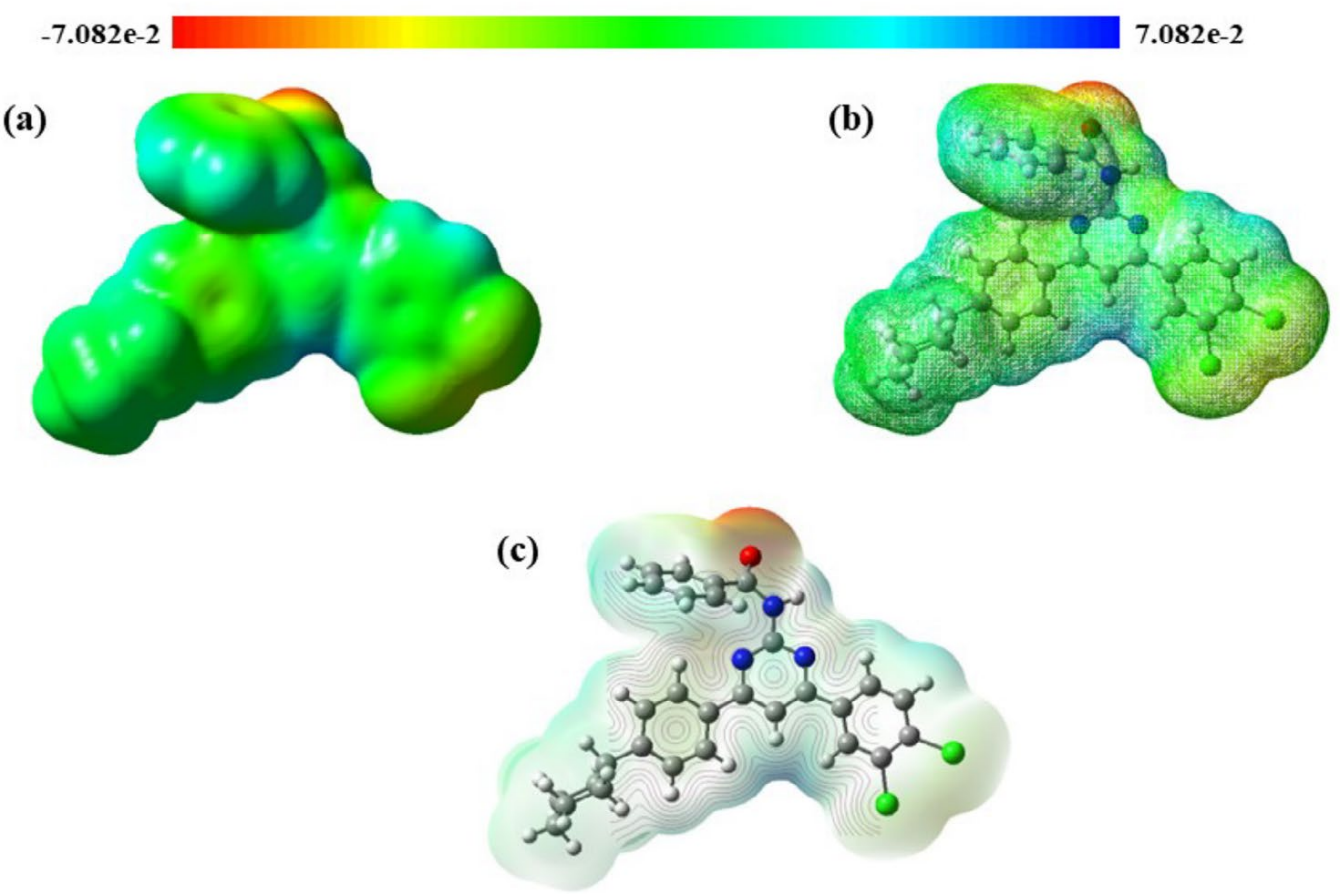
Molecular electrostatic potential (MEP) analysis of **Riv**. (a) MEP surface highlighting electron‐rich (red) and electron‐deficient (green) regions, (b) MEP mesh representation illustrating the three‐dimensional electrostatic potential distribution, and (c) contour map overlay showing potential variations across the molecular surface, aiding in the identification of reactive sites.

Figure 15 illustrates the molecular electrostatic potential (MEP) surface analysis of the compound, providing insights into its electronic distribution and reactivity. Figure 15a presents the MEP surface mapped with potential values, highlighting electron‐rich (negative potential, red regions) and electron‐deficient (positive potential, green regions) zones. These regions correspond to potential sites for nucleophilic and electrophilic interactions, respectively. Figure 15b displays the MEP mesh structure, providing a three‐dimensional representation of the electrostatic potential, further confirming the polarity and distribution of charge across the molecule. Figure 15c overlays contour plots on the molecular structure, showcasing the variation in potential values through color‐coded regions, which provide an in‐depth understanding of the molecular surface's reactive sites. This analysis is crucial for predicting the compound's interaction with EGFR targets and its binding affinity, which is directly linked to its anticancer efficacy. The combined visualization aids in identifying active regions responsible for intermolecular interactions, enhancing **Riv** relevance in anticancer study drug design.

## 3 Comparison With Existing EGFR Inhibitors and Their Potential Advantages in Addressing Clinical Resistance

The synthesized *N*‐Cinnamamide/benzamide‐pyrimidine derivatives, particularly compound **Riv**, demonstrate several significant activities over clinically approved EGFR inhibitors when analysed from both an activity and structural standpoint. Current first‐generation inhibitors such as gefitinib and erlotinib target EGFR^WT^ and activating mutations (e.g., exon 19 deletion or L858R) but rapidly lose efficacy due to the emergence of the T790M gatekeeper mutation. Second‐generation inhibitors like afatinib irreversibly bind EGFR but are associated with dose‐limiting toxicities due to wild‐type EGFR inhibition. Third‐generation inhibitors such as osimertinib selectively target EGFR^L858R/T790M^ mutations with reduced wild‐type activity; however, they are rendered ineffective upon the emergence of the C797S mutation, which disrupts the covalent bond formation at cysteine 797.

In contrast, compound **Riv** exhibited robust enzyme inhibition activity across all four key EGFR variants. Notably, it maintained low IC_50_ values against EGFR^WT^ (00.76 ± 01.11 μM), EGFR^T790M^ (01.06 ± 01.23 μM), EGFR^L858R/T790M^ (01.71 ± 01.31 μM), and EGFR^L858R/T790M/C797S^ (01.02 ± 03.32 μM), demonstrating a broad and balanced inhibitory profile even where osimertinib is compromised. This broad‐spectrum efficacy is also supported by cell‐based antiproliferative assays, where **Riv** outperformed osimertinib in terms of IC_50_ against triple‐mutant cells, indicating better functional activity in a cellular context.

Structurally, **Riv** potency can be attributed to the 3,4‐dichlorophenyl substitution at **R_1_**, which provides electron‐withdrawing character that enhances interaction with the ATP‐binding pocket. The central pyrimidine ring mimics the adenine ring of ATP and forms critical hydrogen bonds with the hinge region. At the same time, the cyclohexyl group extends deeply into a hydrophobic pocket, stabilizing binding through van der Waals interactions, a feature absent in earlier‐generation drugs. The presence of an *N*‐cinnamamide/benzamide side chain improves solubility and pharmacokinetic balance, while also potentially interacting with solvent‐exposed regions or allosteric pockets, increasing the binding surface area.

Moreover, unlike osimertinib, **Riv** was non‐toxic to normal cell lines (e.g., BEAS‐2B, WI‐38), suggesting a superior therapeutic window. Molecular docking studies further demonstrated a U‐shaped conformation within the EGFR active site, mimicking the binding mode of osimertinib but with enhanced hydrophobic and π–π stacking interactions, particularly involving Phe723, Thr854, and Lys745. Therefore, from a medicinal chemistry perspective, **Riv** represents an optimized structural scaffold that balances ATP‐competitive binding, mutation‐resilience (including C797S), and pharmacokinetic safety, meeting several key criteria for a next‐generation EGFR inhibitor. Its multifunctional profile, combining EGFR inhibition, apoptosis induction, and antioxidant activity, underscores its potential clinical advantage in overcoming resistance in NSCLC.

## 4 Conclusion

This study successfully synthesized and characterized a series of 12 novel N‐Cinnamamide/benzamide‐(4‐(substituted phenyl)‐6‐(4‐cyclohexylphenyl)pyrimidin‐2‐yl) derivatives (**Ri–Rxii**) to overcome resistance in EGFR‐driven non‐small cell lung cancer (NSCLC). Among these, compound **Riv** emerged as the most promising candidate, demonstrating potent inhibitory activity against both wild‐type EGFR and its resistant mutants, including the challenging EGFR^L858R/T790M/C797S^ variant. Specifically, compound **Riv** exhibited IC_50_ values of 0.56 ± 01.11 μM for EGFR^WT^, 01.01 ± 0.03 μM for EGFR^T790M^, 0.030 ± 0.003 μM for EGFR^L858R/T790M^, and 01.07 ± 00.02 μM for EGFR^L858R/T790M/C797S^, highlighting its exceptional potency across multiple resistance mutations. Mechanistic studies confirmed its ability to inhibit the EGFR/Akt signaling pathway, while molecular docking and dynamic simulations established its strong and stable interactions with key EGFR residues. Compound **Riv** demonstrated robust binding with amino acid residues Thr854 and Lys745 through hydrogen bonds and π‐π stacking interactions with Phe723, contributing to its superior affinity and selectivity. Additionally, **Riv** effectively induced cell cycle arrest and apoptosis in NSCLC cell lines with minimal toxicity to normal cells. Moreover, in silico ADMET predictions highlighted compound **Riv** favorable pharmacokinetic and toxicity profiles, supporting its potential as a safe and effective anticancer agent. These findings position **Riv** as a strong candidate for further development as a next‐generation therapeutic targeting drug‐resistant NSCLC.

## 5 Material and Methods

### 5.1 Chemistry

The chemicals and solvents required for synthesis were sourced from various manufacturers, including SD Fine Chemicals, Finar, BLD Pharma, Avra Chemicals, TCI, CDH, and Sigma Aldrich. Thin‐layer chromatography (TLC) was employed to ensure chemical homogeneity, using silica gel G (Merck)‐coated plates and solvent systems of toluene/ethyl acetate/formic acid (7:3:1) and ethyl acetate/hexane (2:8). TLC spots were visualized using a UV lamp and an iodine chamber. Uncorrected melting points were determined using open capillary tubes and Hicon melting point equipment. Suction filtration was performed with ashless Whatman No. 1 filter paper. Fourier Transform Infrared Spectroscopy (FTIR) was recorded using a Nicolet MX‐1 FTIR instrument. Proton (^1^H) and carbon (^13^C) NMR spectra were obtained in DMSO solutions at 400 MHz and 100 MHz, respectively, using a Bruker DPX‐400 FT‐NMR spectrophotometer (Bruker Bioscience, Switzerland), with tetramethylsilane [TMS, (CH_3_)4Si] as the internal standard. Signal multiplicities were denoted as s (singlet), brs (broad singlet), d (doublet), dd (double doublet), t (triplet), q (quartet), and m (multiplet). Chemical shift (δ) values are reported in parts per million (ppm). Mass spectra were acquired using an Agilent 6520 Q‐TOF (ESI) mass spectrometer (Agilent Technologies, USA).

#### 5.1.1 General Procedure for the Synthesis (E)‐3‐(Substituted Phenyl)‐1‐(4‐Cyclohexylphenyl)prop‐2‐en‐1‐One (**c1–c6**)

(E)‐3‐(substituted phenyl)‐1‐(4‐cyclohexylphenyl)prop‐2‐en‐1‐one derivatives (**c1–c6**) were synthesized via a Claisen‐Schmidt condensation reaction. Cyclohexyl acetophenone (2 g, 9.88 mmol) and substituted aldehydes (**b1–b6**) (11.86 mmol) were dissolved in 10 mL of ethanol, and 25–30 drops of 30% sodium hydroxide (NaOH) were added to the mixture. The reaction was stirred for 4–5 h at 0°C–5°C. The progress of the reaction was monitored using TLC (30% ethyl acetate: n‐hexane as the mobile phase). Upon completion, the reaction mixture was poured over crushed ice and neutralized with 10% hydrochloric acid (HCl). The resulting solid was filtered, dried, and recrystallized using ethanol to obtain the purified product (Matada et al. 2021; Pal, Matada, Teli, Chawla, et al. 2025).

#### 5.1.2 Synthesis of 4‐(4‐Subsituted Phenyl)‐6‐(4‐Cyclohexylphenyl)pyrimidin‐2‐Amine (**d1–d6**)

A solution of (E)‐3‐(substituted phenyl)‐1‐(4‐cyclohexylphenyl)prop‐2‐en‐1‐one derivatives (**c1–c6**) (2 mmol), guanidine (4 mmol), and 10–15 drops of 30% sodium hydroxide in 10 mL of ethanol was refluxed for 10–12 h. Reaction progress was monitored by TLC using 20% ethyl acetate as the mobile phase. After completion, the reaction mixture was cooled to room temperature, and the precipitate was filtered, washed with 5% dilute HCl, and dried. The obtained product was recrystallized using methanol or column chromatography (10% ethyl acetate) to yield the 4‐(4‐substituted phenyl)‐6‐(4‐cyclohexylphenyl)pyrimidin‐2‐amine derivatives (**d1–d6**) (Pal, Matada, Teli, Akhtar, et al. 2025).

#### 5.1.3 Synthesis of N‐Cinnamamide/Benzamide‐(4‐(Substituted Phenyl)‐6‐(4‐Cyclohexylphenyl)pyrimidin‐2‐yl) (**Ri–Rxii**)

The 4‐(4‐substituted phenyl)‐6‐(4‐cyclohexylphenyl)pyrimidin‐2‐amine derivatives (**d1–d6**) (1 mmol) and potassium carbonate (K_2_CO_3_) (2 mmol) were dissolved in 5–10 mL of dimethylformamide (DMF), and the reaction mixture was stirred for 1–1.5 h at 5°C–10°C, then dropwise, benzoyl chloride or cinnamoyl chloride (1.2 mmol) was added dropwise for 10–20 min. Allowed the reaction mixture to stir at room temperature for another 30 min, and then refluxed at 90°C–100°C for 14–26 h, and the reaction progress was monitored by TLC using a Toluene: Ethyl acetate: Formic acid (TEF) solvent system. Upon completion, the mixture was cooled to room temperature and poured over crushed ice, neutralized with 5% HCl. The resulting solid was filtered, washed thoroughly with water, and dried. The final product was purified by recrystallization using a suitable solvent to obtain the target compounds (**Ri–Rxii**).

##### 5.1.3.1 N‐(4‐(4‐Cyclohexylphenyl)‐6‐(4‐Methoxyphenyl)pyrimidin‐2‐yl)benzamide (**Ri**)

Yield 65%, m.p. 298°C–300°C, *R*_f_: 0.37 (Ethyl acetate:Pet. ether 7:3). IR (KBr cm^−1^): 3280 (N‐H), 3033 (C‐H, Ar), 2840 (C‐H, CH_2_), 1662 (C=O), 1600 (C=C, Ar), 1380 (C‐N), 1241 (C‐O). ^1^HNMR (400 MHz, DMSO) (*δ/*ppm): 9.84 (s, 1H, N‐H), 8.55 (s, 1H, Pyrimidine =CH), 8.02–8.04 (d, 2H, Ar‐H), 7.76–7.78 (d, 2H, Ar‐H), 7.53–7.55 (d, 2H, Ar‐H), 7.46–7.49 (t, 1H, Ar‐H), 7.38–7.40 (d, 2H, Ar‐H), 7.31–7.33 (d, 2H, Ar‐H), 7.00–6.98 (d, 2H, Ar‐H), 3.84 (s, 3H, OCH_3_), 2.85–2.91 (m, 1H, CH), 1.53–1.60 (m, 4H, CH_2_), 1.39–1.44 (m, 2H, CH_2_), 1.23–1.29 (m, 4H, CH_2_). ^13^CNMR (400 MHz, DMSO) (*δ/*ppm): 170.43, 165.60, 161.51, 160.20, 156.43, 145.15, 133.81, 133.13, 131.24, 128.97, 128.12, 127.65, 126.74, 123.81, 111.83, 98.94, 56.89, 42.54, 33.78, 25.95, 23.39. MS: *m*/*z* [M]^+^ for C_30_H_29_N_3_O_2_, calculated: 463.23; observed [M]^+^: 463.39. HPLC purity: 98.41%.

##### 5.1.3.2 N‐(4‐(4‐Cyclohexylphenyl)‐6‐(4‐(Trifluoromethyl)phenyl)pyrimidin‐2‐yl)benzamide (**Rii**)

Yield 69%, m.p. 250°C–252°C, *R*_f_: 0.39 (Ethyl acetate:Pet. ether 7:3). IR (KBr cm^−1^): 3271 (N‐H), 3050 (C‐H, Ar), 2878 (C‐H, CH_2_), 1655 (C=O), 1610 (C=C, Ar), 1380 (C‐N), 1211 (C‐F). ^1^HNMR (400 MHz, DMSO) (*δ/*ppm): 9.57 (s, 1H, N‐H), 8.64 (s, 1H, Pyrimidine =CH), 8.06–8.07 (d, 2H, Ar‐H), 7.80–7.82 (d, 2H, Ar‐H), 7.71–7.73 (d, 2H, Ar‐H), 7.62–7.64 (d, 2H, Ar‐H), 7.50–7.54 (m, 3H, Ar‐H), 7.38–7.40 (d, 2H, Ar‐H), 1.58–1.66 (m, 5H, CH, and CH_2_), 1.47–1.54 (m, 4H, CH_2_), 1.37–1.42 (m, 2H, CH_2_). ^13^CNMR (400 MHz, DMSO) (*δ/*ppm): 170.39, 166.33, 162.20, 156.84, 144.81, 135.43, 133.81, 133.13, 131.35, 130.41, 129.25, 128.31, 127.61, 125.09, 124.32, 123.66, 122.56, 99.41, 42.20, 33.80, 26.03, 23.22. MS: *m*/*z* [M]^+^ for C_30_H_26_F_3_N_3_O, calculated: 501.20; observed [M + 1]^+^: 502.42. HPLC purity: 95.01%.

##### 5.1.3.3 N‐(4‐(4‐Cyclohexylphenyl)‐6‐(2‐Fluorophenyl)pyrimidin‐2‐yl)benzamide (**Riii**)

Yield 62%, m.p. 269°C–271°C, *R*_f_: 0.31 (Ethyl acetate:Pet. Ether 7:3). IR (KBr cm^−1^): 3289 (N‐H), 3025 (C‐H, Ar), 2880 (C‐H, CH_2_), 1659 (C=O), 1605 (C=C, Ar), 1371 (C‐N), 1244 (C‐O). ^1^HNMR (400 MHz, DMSO) (*δ/*ppm): 9.91 (s, 1H, N‐H), 8.56 (s, 1H, Pyrimidine =CH), 7.92–7.93 (d, 2H, Ar‐H), 7.76–7.78 (d, 2H, Ar‐H), 7.23–7.24 (d, 1H, Ar‐H), 7.11–7.15 (m, 3H, Ar‐H), 7.00–7.02 (d, 1H, Ar‐H), 6.71–6.72 (d, 2H, Ar‐H), 6.33–6.35 (d, 2H, Ar‐H), 3.88–3.92 (m, 1H, CH), 2.38–2.41 (m, 4H, CH_2_), 1.77–1.80 (m, 6H, CH_2_). ^13^CNMR (400 MHz, DMSO) (*δ/*ppm): 168.93, 166.30, 162.63, 156.47, 151.26, 143.63, 133.32, 131.71, 130.44, 129.25, 128.06, 127.48, 123.68, 122.37, 121.08, 114.83, 101.03, 43.03, 33.29, 25.62, 24.12. MS: *m*/*z* [M]^+^ for C_29_H_26_FN_3_O, calculated: 451.21; observed [M + 1]^+^: 452.48. HPLC purity: 97.81%.

##### 5.1.3.4 N‐(4‐(4‐Cyclohexylphenyl)‐6‐(3,4‐Dichlorophenyl)pyrimidin‐2‐yl)benzamide (**Riv**)

Yield 63%, m.p. 268°C–270°C, *R*_f_: 0.25 (Ethyl acetate:Pet. ether 7:3). IR (KBr cm^−1^): 3256 (N‐H), 3040 (C‐H, Ar), 2845 (C‐H, CH_2_), 1650 (C=O), 1612 (C=C, Ar), 1387 (C‐N), 1256 (C‐O), 1078 (C‐Cl). ^1^HNMR (400 MHz, DMSO) (*δ/*ppm): 10.51 (s, 1H, N‐H), 9.53 (s, 1H, Pyrimidine =CH), 7.99–8.01 (dd, 2H, Ar‐H), 7.76–7.78 (d, 2H, Ar‐H), 7.56 (s, 1H, Ar‐H), 7.45–7.47 (m, 3H, Ar‐H), 7.38–7.40 (d, 1H, Ar‐H), 7.25–7.27 (d, 2H, Ar‐H), 7.19–7.21 (dd, 1H, Ar‐H), 2.85–2.90 (m, 1H, CH), 1.57–1.68 (m, 4H, CH_2_), 1.44–1.55 (m, 6H, CH_2_). ^13^CNMR (400 MHz, DMSO) (*δ/*ppm): 170.84, 165.34, 162.41, 156.71, 145.43, 133.81, 133.43, 133.13, 132.82, 132.52, 132.15, 130.41, 128.96, 128.52, 128.28, 127.65, 127.02, 124.13, 98.82, 43.20, 34.15, 26.05, 23.17. HR‐MS: *m*/*z* [M]^+^ for C_29_H_25_Cl_2_N_3_O, calculated: 501.14; observed [M + 1]^+^: 502.2503. HPLC purity: 97.38%.

##### 5.1.3.5 N‐(4‐(3‐Bromo‐4‐Methoxyphenyl)‐6‐(4‐Cyclohexylphenyl)pyrimidin‐2‐yl)benzamide (**Rv**)

Yield 60%, m.p. 272°C–274°C, *R*_f_: 0.29 (Ethyl acetate:Pet. ether 7:3). IR (KBr cm^−1^): 3277 (N‐H), 3018 (C‐H, Ar), 2840 (C‐H, CH_2_), 1666 (C=O), 1601 (C=C, Ar), 1387 (C‐N), 1290 (C‐O), 1044 (C‐Br). ^1^HNMR (400 MHz, DMSO) (*δ/*ppm): 9.96 (s, 1H, N‐H), 8.57 (s, 1H, Pyrimidine =CH), 8.02–8.04 (d, 2H, Ar‐H), 7.76–7.78 (d, 2H, Ar‐H), 7.64–7.66 (d, 2H, Ar‐H), 7.58–7.61 (t, 1H, Ar‐H), 7.51–7.53 (d, 2H, Ar‐H), 7.42–7.44 (d, 1H, Ar‐H), 7.39 (s, 1H, A‐H), 6.94–6.97 (d, 1H, Ar‐H), 3.91 (s, 3H, OCH_3_), 2.40–2.47 (m, 1H, CH), 1.70–1.81 (m, 4H, CH_2_), 1.46–1.53 (m, 4H, CH_2_), 1.30–1.36 (m, 2H, CH_2_). ^13^CNMR (400 MHz, DMSO) (*δ/*ppm): 168.12, 165.22, 161.84, 155.48, 152.36, 145.73, 133.75, 133.43, 132.16, 131.23, 128.83, 128.16, 127.59, 127.12, 126.82, 125.22, 112.73, 111.80, 98.86, 55.13, 42.69, 33.91, 25.70, 24.82. MS: *m*/*z* [M]^+^ for C_30_H_28_BrN_3_O_2_, calculated: 541.14; observed [M + 1]^+^: 542.14. HPLC purity: 95.18%.

##### 5.1.3.6 N‐(4‐(4‐Cyclohexylphenyl)‐6‐(4‐Ethoxy‐3‐Methoxyphenyl)pyrimidin‐2‐yl)benzamide (**Rvi**)

Yield 66%, m.p. 245°C–247°C, *R*_f_: 0.35 (Ethyl acetate:Pet. ether 7:3). IR (KBr cm^−1^): 3289 (N‐H), 3050 (C‐H, Ar), 2900 (C‐H, CH_2_), 1661 (C=O), 1625 (C=C, Ar), 1359 (C‐N), 1264 (C‐O). ^1^HNMR (400 MHz, DMSO) (*δ/*ppm): 10.05 (s, 1H, N‐H), 8.78 (s, 1H, Pyrimidine =CH), 8.04–8.06 (d, 2H, Ar‐H), 7.75–7.78 (d, 2H, Ar‐H), 7.61–7.63 (t, 1H, Ar‐H), 7.52–7.55 (d, 2H, Ar‐H), 7.51–7.53 (d, 2H, Ar‐H), 7.29 (s, 1H, Ar‐H), 7.21–7.23 (d, 1H, Ar‐H), 6.91–6.94 (d, 1H, Ar‐H), 3.95–4.01 (m, 2H, OCH_2_), 3.78 (s, 3H, OCH_3_), 2.72–2.79 (m, 1H, CH), 1.46–1.54 (m, 4H, CH_2_), 1.38–1.42 (t, 3H, CH_3_), 1.30–1.34 (m, 2H, CH_2_). ^13^CNMR (400 MHz, DMSO) (*δ/*ppm): 167.81, 163.18, 161.22, 156.40, 148.86, 148.60, 145.29, 133.48, 133.10, 132.18, 129.33, 128.84, 128.55, 127.39, 125.41, 120.44, 111.10, 109.25, 98.83, 63.86, 56.22, 42.26, 33.21, 26.00, 24.54, 17.29. MS: *m*/*z* [M]^+^ for C_32_H_33_N_3_O_3_, calculated: 507.25; observed [M + 1]^+^: 508.26. HPLC purity: 94.86%.

##### 5.1.3.7 N‐(4‐(4‐Cyclohexylphenyl)‐6‐(4‐Methoxyphenyl)pyrimidin‐2‐yl)cinnamamide (**Rvii**)

Yield 72%, m.p. 276°C–278°C, *R*_f_: 0.31 (Ethyl acetate:Pet. ether 7:3). IR (KBr cm^−1^): 3302 (N‐H), 3030 (C‐H, Ar), 2872 (C‐H, CH_2_), 1649 (C=O), 1628 (Vinyl C=C), 1604 (C=C, Ar), 1344 (C‐N), 1290 (C‐O). ^1^HNMR (400 MHz, DMSO) (*δ/*ppm): 9.99 (s, 1H, N‐H), 8.55 (s, 1H, Pyrimidine =CH), 7.87–7.89 (d, 2H, Ar‐H), 7.71–7.73 (d, 2H, Ar‐H), 7.64–7.66 (d, 1H, =CH‐Ar), 7.59–7.61 (d, 2H, Ar‐H), 7.48–7.51 (d, 2H, Ar‐H), 7.38–7.40 (d, 2H, Ar‐H), 7.32–7.37 (t, 1H, Ar‐H), 7.05–7.08 (d, 2H, Ar‐H), 6.95–6.97 (d, 1H, O=C‐CH=), 3.73 (s, 3H, OCH_3_), 2.80–2.89 (m, 1H, CH), 1.65–1.85 (m, 4H, CH_2_), 1.47–1.56 (m, 4H, CH_2_), 1.41–1.45 (m, 2H, CH_2_). ^13^CNMR (400 MHz, DMSO) (*δ/*ppm): 168.66, 165.28, 162.02, 158.23, 157.26, 145.23, 140.39, 134.25, 133.32, 128.96, 128.53, 128.10, 127.86, 126.59, 125.40, 118.26, 114.82, 101.05, 55.81, 42.33, 34.66, 25.74, 24.45. MS: *m*/*z* [M]^+^ for C_32_H_31_N_3_O_2_, calculated: 489.24; observed [M + 1]^+^: 490.24. HPLC purity: 97.06%.

##### 5.1.3.8 N‐(4‐(4‐Cyclohexylphenyl)‐6‐(4‐(Trifluoromethyl)phenyl)pyrimidin‐2‐yl)cinnamamide (**Rviii**)

Yield 75%, m.p. 266°C–268°C, *R*_f_: 0.30 (Ethyl acetate:Pet. Ether 7:3). IR (KBr cm^−1^): 3278 (N‐H), 3043 (C‐H, Ar), 2919 (C‐H, CH_2_), 1655 (C=O), 1625 (Vinyl C=C), 1595 (C=C, Ar), 1356 (C‐N), 1247 (C‐O). ^1^HNMR (400 MHz, DMSO) (*δ/*ppm): 10.25 (s, 1H, N‐H), 8.48 (s, 1H, Pyrimidine =CH), 7.87–7.89 (d, 1H, Ar‐H), 7.76–7.78 (d, 2H, Ar‐H), 7.65–7.66 (d, 2H, Ar‐H), 7.54–7.58 (m, 3H, Ar‐H), 7.45–7.47 (t, 1H, Ar‐H), 7.38–7.40 (d, 2H, Ar‐H), 7.27–7.30 (t, 1H, Ar‐H), 7.16–7.20 (t, 1H, Ar‐H), 6.89–6.92 (d, 1H, O=C‐CH=), 6.57–6.61 (d, 1H, =CH‐Ar), 2.90–2.93 (m, 1H, CH), 1.57–1.68 (m, 4H, CH_2_), 1.51–1.56 (m, 4H, CH_2_), 1.45–1.50 (m, 2H, CH_2_). ^13^CNMR (400 MHz, DMSO) (*δ/*ppm): 172.37, 165.80, 162.41, 155.92, 144.84, 140.88, 138.13, 136.13, 133.81, 131.41, 128.96, 128.62, 128.01, 127.35, 126.33, 125.62, 124.92, 123.70, 116.26, 99.73, 41.87, 33.85, 25.62, 23.33. HR‐MS: *m*/*z* [M]^+^ for C_32_H_28_F_3_N_3_O, calculated: 527.22; observed [M + 1]^+^: 428.1143. HPLC purity: 96.66%.

##### 5.1.3.9 N‐(4‐(4‐Cyclohexylphenyl)‐6‐(2‐Fluorophenyl)pyrimidin‐2‐yl)cinnamamide (**Rix**)

Yield 78%, m.p. 286°C–288°C, *R*_f_: 0.38 (Ethyl acetate:Pet. ether 7:3). IR (KBr cm^−1^): 3322 (N‐H), 3036 (C‐H, Ar), 2879 (C‐H, CH_2_), 1662 (C=O), 1631 (Vinyl C=C), 1605 (C=C, Ar), 1337 (C‐N), 1277 (C‐O). ^1^HNMR (400 MHz, DMSO) (*δ/*ppm): 9.51 (s, 1H, N‐H), 8.48 (s, 1H, Pyrimidine =CH), 7.76–7.78 (d, 2H, Ar‐H), 7.62 (s, 1H, Ar‐H), 7.50–7.56 (m, 5H, Ar‐H), 7.42–7.45 (d, 1H, Ar‐H), 7.37–7.39 (d, 2H, Ar‐H), 7.25–7.27 (d, 1H, Ar‐H), 7.19–7.21 (dd, 1H, O=C‐CH=), 6.57–6.61 (d, 1H, =CH‐Ar), 2.86–2.93 (m, 1H, CH), 1.49–1.55 (m, 4H, CH_2_), 1.37–1.43 (m, 4H, CH_2_), 1.22–1.30 (m, 2H, CH_2_). ^13^CNMR (400 MHz, DMSO) (*δ/*ppm): 171.93, 165.83, 161.62, 155.78, 154.15, 144.82, 141.71, 136.13, 131.11, 129.89, 129.39, 128.97, 128.15, 124.46, 123.20, 117.15, 113.51, 101.01, 42.52, 34.60, 25.83, 25.10. MS: *m*/*z* [M]^+^ for C_31_H_28_FN_3_O, calculated: 477.22; observed [M + 1]^+^: 478.21. HPLC purity: 98.02%.

##### 5.1.3.10 N‐(4‐(4‐Cyclohexylphenyl)‐6‐(3,4‐Dichlorophenyl)pyrimidin‐2‐yl)cinnamamide (**Rx**)

Yield 70%, m.p. 262°C–264°C, *R*_f_: 0.37 (Ethyl acetate:Pet. ether 7:3). IR (KBr cm^−1^): 3270 (N‐H), 3050 (C‐H, Ar), 2845 (C‐H, CH_2_), 1644 (C=O), 1627 (Vinyl C=C), 1590 (C=C, Ar), 1347 (C‐N), 1250 (C‐O). ^1^HNMR (400 MHz, DMSO) (*δ/*ppm): 10.08 (s, 1H, N‐H), 8.60 (s, 1H, Pyrimidine =CH), 7.94 (s, 1H, Ar‐H), 7.82–7.85 (d, 1H, Ar‐H), 7.76–7.78 (d, 2H, Ar‐H), 7.63–7.65 (d, 1H, =CH‐Ar), 7.58–7.60 (d, 2H, Ar‐H), 7.52–7.53 (d, 1H, Ar‐H), 7.49–7.50 (d, 2H, Ar‐H), 7.40–7.42 (d, 2H, Ar‐H), 7.32–7.37 (t, 1H, Ar‐H), 6.93–6.95 (d, 1H, O=C‐CH=), 2.76–2.83 (m, 1H, CH), 1.75–1.82 (m, 4H, CH_2_), 1.44–1.50 (m, 4H, CH_2_), 1.31–1.39 (m, 2H, CH_2_). ^13^CNMR (400 MHz, DMSO) (*δ/*ppm): 172.41, 165.43, 162.31, 156.28, 143.73, 140.86, 136.10, 134.37, 133.53, 132.75, 132.18, 130.78, 130.12, 129.39, 128.97, 128.18, 127.22, 126.54, 124.90, 116.39, 99.63, 41.93, 34.15, 25.66, 24.81. MS: *m*/*z* [M]^+^ for C_31_H_27_Cl_2_N_3_O, calculated: 527.15; observed [M + 1]^+^: 528.15. HPLC purity: 94.20%.

##### 5.1.3.11 N‐(4‐(3‐Bromo‐4‐Methoxyphenyl)‐6‐(4‐Cyclohexylphenyl)pyrimidin‐2‐yl)cinnamamide (**Rxi**)

Yield 78%, m.p. 268°C–271°C, *R*_f_: 0.35 (Ethyl acetate:Pet. ether 7:3). IR (KBr cm^−1^): 3350 (N‐H), 3022 (C‐H, Ar), 2845 (C‐H, CH_2_), 1640 (C=O), 1625 (Vinyl C=C), 1600 (C=C, Ar), 1347 (C‐N), 1225 (C‐O). ^1^HNMR (400 MHz, DMSO) (*δ/*ppm): 10.27 (s, 1H, N‐H), 8.71 (s, 1H, Pyrimidine =CH), 7.74–7.76 (d, 2H, Ar‐H), 7.64–7.66 (d, 1H, =CH‐Ar), 7.59–7.61 (d, 2H, Ar‐H), 7.51–7.53 (d, 2H, Ar‐H), 7.43–7.45 (d, 1H, Ar‐H), 7.38–7.40 (d, 2H, Ar‐H), 7.36 (s, 1H, Ar‐H), 7.30–7.34 (t, 1H, Ar‐H), 6.96–6.98 (d, 1H, Ar‐H), 6.89–6.91 (d, 1H, O=C‐CH=), 3.88 (s, 3H, OCH_3_), 2.72–2.84 (m, 1H, CH), 1.65–1.85 (m, 4H, CH_2_), 1.46–1.55 (m, 4H, CH_2_), 1.28–1.33 (m, 2H, CH_2_). ^13^CNMR (400 MHz, DMSO) (*δ/*ppm): 166.86, 164.26, 162.37, 156.23, 154.29, 146.24, 140.36, 134.27, 133.15, 132.86, 128.80, 128.55, 127.69, 127.10, 126.94, 125.91, 118.26, 112.53, 111.87, 99.82, 56.37, 42.23, 33.78, 25.23, 26.11. MS: *m*/*z* [M]^+^ for C_32_H_30_BrN_3_O_2_, calculated: 567.15; observed [M + 1]^+^: 568.52. HPLC purity: 94.58%.

##### 5.1.3.12 N‐(4‐(4‐Cyclohexylphenyl)‐6‐(4‐Ethoxy‐3‐Methoxyphenyl)pyrimidin‐2‐yl)cinnamamide (**Rxii**)

Yield 79%, m.p. 300°C–302°C, *R*_f_: 0.27 (Ethyl acetate:Pet. ether 7:3). IR (KBr cm^−1^): 3248 (N‐H), 3040 (C‐H, Ar), 2936 (C‐H, CH_2_), 1650 (C=O), 1633 (Vinyl C=C), 1597 (C=C, Ar), 1336 (C‐N), 1225 (C‐O). ^1^HNMR (400 MHz, DMSO) (*δ/*ppm): 10.21 (s, 1H, N‐H), 8.76 (s, 1H, Pyrimidine =CH), 7.86–7.88 (d, 1H, Ar‐H), 7.76–7.78 (d, 2H, Ar‐H), 7.61–7.63 (d, 1H, =CH‐Ar), 7.51–7.53 (d, 2H, Ar‐H), 7.36–7.39 (d, 2H, Ar‐H), 7.28–7.31 (t, 1H, Ar‐H), 7.25 (s, 1H, Ar‐H), 7.20–7.22 (d, 1H, Ar‐H), 6.93–6.95 (d, 1H, Ar‐H), 6.86–6.88 (d, 1H, O=C‐CH=), 3.92–3.98 (m, 2H, OCH_2_), 3.77 (s, 3H, OCH_3_), 2.72–2.79 (m, 1H, CH), 1.63–1.82 (m, 4H, CH_2_), 1.46–1.54 (m, 4H, CH_2_), 1.38–1.42 (t, 3H, CH_3_), 1.29–1.34 (m, 2H, CH_2_). ^13^CNMR (400 MHz, DMSO) (*δ/*ppm): 167.72, 164.25, 161.82, 156.33, 148.96, 148.20, 145.27, 140.26, 135.11, 133.27, 129.36, 128.50, 128.24, 127.66, 126.83, 124.96, 120.41, 118.82, 113.26, 108.81, 98.60, 62.88, 56.13, 43.14, 33.85, 25.87, 24.63, 16.74. MS: *m*/*z* [M]^+^ for C_34_H_35_N_3_O_3_, calculated: 533.27; observed [M + 1]^+^: 534.27. HPLC purity: 95.70%.

### 5.2 Pharmacological Activities

#### 5.2.1 In Vitro Antiproliferative Activity

The antiproliferative activity of the synthesized compounds was assessed using a panel of non‐small cell lung cancer (NSCLC) cell lines, including A549 (EGFR^WT^), NCI‐H1975 (EGFR^L858R/T790M^), HCC827 (EGFR^Del E746‐A750^), and NCI‐H1975 (EGFR^L858R/T790M/C797S^). Standard tyrosine kinase inhibitors—erlotinib, gefitinib, and osimertinib—served as positive controls to benchmark the efficacy of the test compounds. The antiproliferative effects were evaluated using the MTT assay, following a methodology adapted from Gaber and colleagues. Briefly, absorbance at 490 nm was measured for each well using an automated ELISA reader (TECAN, CHE), providing an indirect measure of cell viability based on metabolic activity. To further evaluate selectivity, the in vitro anticancer activity of the compounds was also tested on non‐cancerous human cell lines, including T293, WI‐38, and BEAS‐2B. These assays were performed following previously established protocols. Half‐maximal inhibitory concentration (IC_50_) values were determined through nonlinear regression analysis using GraphPad Prism software (Version 5). The results are presented as the means of at least three independent experiments, each performed in triplicate. Statistical analysis was conducted using one‐way analysis of variance (ANOVA), with *p*‐values < 0.05 considered indicative of statistically significant differences (Pawara et al. 2021; Matada et al. 2021; Gupta et al. 2016).

#### 5.2.2 EGFR^WT^ , EGFR^T790M^ , EGFR^L858R^ ^/T790M^, and EGFR^L858R^ ^/T790M/C797S^ Kinase Inhibitory Assay

The inhibitory activity of the selected derivatives against EGFR^WT^, EGFR^T790M^, EGFR^L858R/T790M^, and EGFR^L858R/T790M/C797S^ was assessed in vitro using a homogeneous time‐resolved fluorescence (HTRF) assay. The EGFR^WT^, EGFR^T790M^, EGFR^L858R/T790M^, and EGFR^L858R/T790M/C797S^ ATP were procured from Sigma. The assay was initiated by incubating EGFR^WT^, EGFR^T790M^, EGFR^L858R/T790M^, and EGFR^L858R/T790M/C797S^ with their respective substrates in the enzymatic buffer for 5 min at room temperature. Subsequently, ATP (final concentration: 1.65 μM) was added to the reaction mixture, and the enzymatic reaction was allowed to proceed for 30 min. The addition of EDTA‐containing detection reagents terminated the reaction. Following a 1‐h detection phase, the compounds' inhibitory concentrations (IC_50_ values) were determined using nonlinear regression analysis performed in GraphPad Prism software (Version 5.0). All concentrations were tested in triplicate, with experiments conducted independently on three separate occasions to ensure the reliability and reproducibility of the results (Pawara et al. 2021; Matada et al. 2021; Xia et al. 2021).

#### 5.2.3 Cell Cycle Analysis

The Six‐well plates were seeded with 10^6^ NCI‐H1975 cells per well and incubated for 18 h. Different quantities of **Riii** and **Riv** were added to the cells and then incubated for the following 24 h. They were trypsinized to harvest the cells from the plates after being cleaned with PBS. Following PBS washing, cells were fixed for 30 min in ice‐cold 70% ethanol. After 10 min of resuspending the cells in PBS containing RNase A, 50 mL of PI (500 mg/mL) was added to stain the cellular DNA. The staining procedure lasted 30 min at 4°C in the dark. Using flow cytometry (BD Biosciences), the DNA content of the labeled cells was examined, and the distribution of the cycles was measured. Two separate times were used for each experiment (Pawara et al. 2021).

#### 5.2.4 Apoptosis Assay

In a 6‐well plate, 10^6^ cells per well were planted, and the cells were incubated for 18 h. After being exposed to several doses of **Riii** and **Riv** for 24 h, the cells were collected using trypsin. An annexin V‐FITC Apoptosis kit (SIGMA ALDRICH) was used to perform flow cytometry on cell pellets after twice washing with PBS to assess cellular apoptosis or necrosis. The assay's methodology was used following the guidelines provided by the kit manufacturer (Pawara et al. 2021).

#### 5.2.5 Antioxidant Activity

The in vitro antioxidant activity of the titled compounds was evaluated using the DPPH (1,1‐diphenyl‐2‐picrylhydrazyl), H_2_O_2_ (hydrogen peroxide), and 2,2′‐Azinobis‐3‐ethylbenzthiazoline‐6‐sulfonic acid (ABTS˙^+^) scavenging assay techniques using ascorbic acid as a reference drug (Shahab et al. 2019).

##### 5.2.5.1 DPPH Method

The analysis of antioxidant activity using the DPPH assay relies on the principle that hydrogen‐donating molecules act as antioxidants, enabling the detection of compounds that function as radical scavengers. The antioxidant activity is directly proportional to the reduction of DPPH in the test samples. DPPH, a stable free radical, exhibits a strong absorption maximum at 517 nm (appearing purple). When an antioxidant donates hydrogen, the DPPH radical is reduced, causing a color change from purple to yellow, which signifies the formation of the reduced DPPH. This reaction is stoichiometric, corresponding to the number of hydrogen atoms accepted. Thus, the antioxidant activity can be quantified by measuring the decrease in UV absorbance at 517 nm (Pal et al. 2021).

A fresh DPPH solution (0.1 mM in methanol) was prepared and stored in the dark for 2 h. For each sample, four concentrations (5, 10, 15, and 20 μg/mL) were prepared. To each test tube, 2 mL of the respective sample concentration was added, followed by 2 mL of the freshly prepared DPPH solution. The mixtures were thoroughly mixed and incubated at room temperature for 30 min. After incubation, the UV absorbance was measured at 517 nm to evaluate the antioxidant activity (Shahab et al. 2019).

##### 5.2.5.2 H_2_O_2_ Method

The novel synthesized cyclohexyl‐pyrimidine derivatives were also assessed using the hydrogen peroxide method. Different concentrations (5, 10, 15, and 20 μg/mL) in methanol were transferred into test tubes, and the volume was adjusted to 0.4 mL with 50 mM phosphate buffer (pH 7.4). Next, 0.6 mL of a 2 mM H_2_O_2_ solution was added. The reaction mixture was vortexed, and after 10 min, the absorbance was measured at 230 nm. Ascorbic acid was used as the positive control (Shahab et al. 2019; Bedini et al. 2018). The H_2_O_2_ scavenging activity of the compounds was calculated using the following equation:

$$H_{2}O_{2}\;\text{scavenging activity percentage}=\left[\left(A_{0}-A_{1}\right)/A_{0}\right]\times 100$$

where *A*_0_ = absorbance of control, *A*_1_ = Absorbance of a sample.

##### 5.2.5.3 ABTS Radical Scavenging Activity

The ABTS assay was used to evaluate the antioxidant potential of biological fluids, tissues, and both natural and synthetic complexes. Following a previously established protocol, the formation of the ABTS+ radical cation was induced by metmyoglobin and hydrogen peroxide. A 7 μM solution of ABTS was allowed to react with 2.45 μM potassium persulfate in the dark for 12 h to generate the dark‐colored ABTS radical cations. The ABTS solution used in the assay was prepared by diluting it with 50% methanol to achieve an absorbance of approximately 0.70 at 745 nm.

To assess ABTS radical quenching, 1.0 mL of the ABTS working solution was mixed with 100 μL of the test sample. The decrease in absorbance was measured precisely after 1 min, again at the 3‐min mark, and a final reading was taken at the 6‐min mark (Biskup et al. 2013). The following formula was applied to calculate percentage inhibition:

$$\begin{array}{c} \text{ABTS radical scavenging activity}\;\left(\%\right)\\ =\left[\left(A_{734}\text{blank}-A_{734}\text{sample}\right)/A_{734}\text{blank}\right]\times 100. \end{array}$$

where *A*_734_blank and *A*_734_sample represent the absorbance of the blank and test sample at 734 nm, respectively.

### 5.3 Evaluation of Nitric Oxide Release

The determination of nitric oxide (NO) release was carried out following a protocol similar to that reported by Abadi et al. (Abadi et al. 2005). The reaction mixtures of all compounds were treated with 250 μL of Griess reagent, and the final volume was adjusted to 1 mL. The absorbance was recorded at 540 nm at time‐dependent intervals of 5, 10, 15, 20, 25, 30, 35, 40, 45, and 50 min (Abadi et al. 2005; Alshazly et al. 2026). The Griess reagent was prepared by dissolving sulfanilamide (4 g) and *N*‐naphthylethylenediamine dihydrochloride (0.2 g) in 85% phosphoric acid (10 mL) and diluting with distilled water to a final volume of 100 mL (Abadi et al. 2005).

### 5.4 In Silico Studies

#### 5.4.1 In Silico EGFR^WT^ , EGFR^T790M^ and ^EGFRL858R/T790M/C797S^ Inhibition Property

The inhibition constant (*Ki*) for *EGFR* ^*WT*^, *EGFR* ^*T790M*^, *and EGFR* ^*L858R/T790M/C797S*^ were calculated using a similar protocol reported by Pal et al. (2022) and Ortiz et al. (2019) using the formula:

$$\mathrm{Ki}=\exp\left(\Delta G/\mathrm{RT}\right)$$

where: *R* = universal gas constant (1.985 × 10^–3^ kcal/mol/K); *T* = temperature (298.15 K).

#### 5.4.2 In Silico Prediction of ADMET

A computational study of ADME properties of the title compounds was predicted by ADMETlab 3.0 (https://admetlab3.scbdd.com/server/evaluationCal) and ProTox 3.0 (https://tox.charite.de/protox3/) online software (Banerjee et al. 2024; Fu et al. 2024).

#### 5.4.3 Docking Studies

To ascertain the interaction mode and binding energy of the title compounds, molecular docking research was performed using EGFR^WT^, EGFR^T790M/L858R^, and EGFR^L858R/T790M/C797S^, which were retrieved from the protein data bank and had PDB IDs of 4I23 (Engel et al. 2015), 3W2O (Sogabe et al. 2013), and 6LUD (Kashima et al. 2020), respectively. AutoDockTools was used to prepare all input files (version 4.2.6). ChemDraw version 23.1.1 was used to create the 2D structure for each chemical (**Ri–Rxii**). The energies were reduced and converted to a pdb file using Chem3D Extreme (version 23.1.1). For the EGFR^WT^, EGFR^T790M/L858R^, and EGFR^L858R/T790M/C797S^ enzyme input file, non‐polar hydrogens were merged, and the partial atomic charge was calculated using the Kollman approach. The pdbqt file format was used to store the created file. AutoDock was used to produce a grid map with grid boxes of 24 × 24 × 24 Å (EGFR^WT^), 26 × 26 × 25 Å (EGFR^T790M/L858R^), and 44 × 34 × 38 Å (EGFR^L858R/T790M/C797S^) points. The docking procedure was completed using AutoDock vina 1.3.5. PyMol Tcl version 1.1. The results were recorded in terms of binding energies (Δ*G*), and for the calculation of ΔG, AutoDock Vina uses a semi‐empirical scoring function that combines several energy terms:

$$\begin{array}{c} \Delta G_{\text{binding}}=\Delta G_{\text{gauss}}+\Delta G_{\text{repulsion}}+\Delta G_{\text{hydrophobic}}\\ \;+\Delta G_{\text{hydrogen bonding}}+\Delta G_{\text{torsion}} \end{array}$$

where Δ*G*_gauss_: Approximate steric interactions (both attractive and repulsive parts); Δ*G*_repulsion_: Penalizes atomic overlap; Δ*G*_hydrophobic_: Accounts for desolvation and nonpolar interactions; Δ*G*_hydrogen bonding_: Captures favorable H‐bond formation; Δ*G*_torsion_: Penalizes ligand flexibility (entropy loss upon binding).

Discovery Studio visualizer version 17.222 was used for the analysis and visualization (Pal et al. 2022; Pal, Kumar, et al. 2023).

#### 5.4.4 Molecular Dynamic Simulation Studies

Molecular dynamics (MD) simulations were conducted using the Desmond software, an explicit solvent MD program, along with the OPLS 2005 force field, to study the promising compound **Riv** with a favorable docking score in complex with EGFR^L858R/T790M/C797S^ (PDB ID: 6LUD) (Zhu et al. 2014). The protein‐ligand complex was prepared using the Protein Preparation Wizard, with the SPC (simple point charge) water model and an orthorhombic box shape. To set the ionic strength, sodium chloride at a physiological concentration of approximately 0.15 M was added between the protein atoms and the simulation box, within a 10 Å buffer region. Energy minimization was performed to relax the system to a local energy minimum. Following this, the system was subjected to a 100 ns molecular dynamics (MD) simulation using the OPLS_2005 force field. The MD simulation employed the Nose‐Hoover chain thermostat at 300 K, a Coulombic cutoff of 0.9 nm, and the Martyna‐Tobias‐Klein barostat at 1.01325 bar. All other parameters were set to their default values. The resulting MD simulation trajectories, focusing on ligand‐receptor interactions, were analyzed using the Simulation Interaction Diagram (SID) (Zhu et al. 2014; Vora et al. 2020).

#### 5.4.5 DFT Analysis

DFT analysis was performed by Xing et al.'s procedure. Density functional theory (DFT) is a well‐respected computational method because of its capacity to accurately predict molecular properties. The DFT calculations were performed using Gaussian 16 software and the B3LYP functional with the def2‐SVP basis set. Justification is provided by the reliable geometry optimizations offered by the selected functional and basis set. The molecular electrostatic potential (MEP) surface was also analyzed in this study. Additionally, the energies of the lowest unoccupied molecular orbital (_E_LUMO) and the highest occupied molecular orbital (_E_HOMO) were utilized to calculate key chemical reactivity and stability descriptors of the molecules. These include the energy gap (∆*E*), ionization potential (IP), electron affinity (EA), electronegativity (*χ*), chemical potential (*μ*), chemical hardness (*η*), softness (S), and the electrophilicity index (*ω*) (Althobaiti et al. 2023).

## Author Contributions

**Gurubasavaraja Swamy Purawarga Matada:** supervision, funding acquisition, visualization. **Manjushree BV:** methodology, writing – review and editing, resources. **Anguraj Moulishankar:** validation, resources, writing – review and editing. **Viney Chawla:** methodology, resources. **Ghanshyam Teli:** writing – review and editing, formal analysis, software. **Rohit Pal:** conceptualization, methodology, data curation, formal analysis, supervision, project administration, visualization, validation, writing – review and editing, writing – original draft. **Pooja A. Chawla:** resources.

## Funding

This research was financially supported by the Indian Council of Medical Research (ICMR), New Delhi, under the programs No. 5/13/12/2020/NCD‐III, No. 5/13/79/2020/NCD‐III, and No. IIRPSG‐2024‐01‐01202.

## Conflicts of Interest

The authors declare no conflicts of interest.

## Supporting information


**Figure S1:** Displays the Selectivity Index (SI) values for the most promising compounds (**Riii**, **Riv**, and **Rx**) across cancer cell lines. Compound **Riii** stands out with exceptionally high selectivity for HCC827. Compounds **Riv** and **Rx** also show strong selectivity for HCC827, making them promising candidates.
**Table S1:** Docking score of titled compounds.

## Data Availability

The data that supports the findings of this study are available in the Supporting Information of this article.
