# Supplementary material for: Rational Design, Synthesis, Biological Evaluation, and In Silico Study of N‐Cinnamamide/Benzamide‐(4‐(Substituted Phenyl)‐6‐(4‐Cyclohexylphenyl)pyrimidin‐2‐yl) Derivatives as EGFR Inhibitors Targeting EGFRWT , EGFRT790M , and EGFRL858R /T790M/C797S in NSCLC
Source: Chem Biol Drug Des. 2026 Sep 8;108(3):e70390. doi: 10.1111/cbdd.70390 (PMC13554467; doi:10.1111/cbdd.70390)
Supplement: Supplementary file 1 [file CBDD-108-e70390-s001.docx]

**Supplementary data**

**Rational Design, Synthesis, Biological Evaluation, and *In-silico* study of *N*- Cinnamamide/Benzamide-(4-(substituted phenyl)-6-(4-cyclohexylphenyl)pyrimidin-2-yl) derivatives as EGFR Inhibitors Targeting EGFR^WT^, EGFR^T790M^, and EGFRL858R/T790M/C797S in NSCLC**

Rohit Pal^1*^, Gurubasavaraja Swamy Purawarga Matada^1*^, Ghanshyam Teli^2^, Ghanshyam Teli^2^, Manjushree B V^1^, Viney Chawla^3^, Pooja A. Chawla^3^

*^1^Authors Affiliation: ^1^Integrated Drug Discovery Centre, Department of Pharmaceutical Chemistry, Acharya & BM Reddy College of Pharmacy, Bengaluru 560107, Karnataka, India*

*^2^School of Pharmacy, Sangam University, Atoon, Bhilwara, 311001, Rajasthan, India*

*^3^University Institute of Pharmaceutical Sciences and Research, Baba Farid University of Health Sciences, Faridkot, Punjab, 151203, India*

**Corresponding author*

**Rohit Pal**

Integrated Drug Discovery Centre, Department of Pharmaceutical Chemistry, Acharya & BM Reddy College of Pharmacy, Bengaluru 560107, Karnataka, India

Email ID: [rohitpal.rp096@gmail.com](mailto:rohitpal.rp096@gmail.com)

ORCID: <https://orcid.org/0000-0002-2763-5757>

**Corresponding author*

***Prof. (Dr.) Gurubasavaraja Swamy Purawarga Matada***

Integrated Drug Discovery Centre, Department of Pharmaceutical Chemistry, Acharya & BM Reddy College of Pharmacy, Bengaluru 560107, Karnataka, India

E-mail: [gurubasavarajaswamy@gmail.com](mailto:gurubasavarajaswamy@gmail.com)

- *Selectivity of analysis of title compounds*

**Fig. S1**: Displays the Selectivity Index (SI) values for the most promising compounds (**Riii**, **Riv**, and **Rx**) across cancer cell lines. Compound **Riii** stands out

with exceptionally high selectivity for HCC827. Compounds **Riv** and **Rx** also show strong selectivity for HCC827, making them promising candidates.

- Compares SI values across normal cell lines for the top compounds. For each compound, the highest SI values are consistently associated with MCF-10A and BEAS-2B, further emphasizing their selectivity.
  - *Docking analysis*

Molecular docking studies were performed to evaluate the binding affinity of the synthesized compounds (**Ri-Rxii**) against three EGFR kinase variants: EGFRWT (PDB ID: 4I23), EGFRT790M/L858R (PDB ID: 3W2O), and EGFRL858R/T790M/C797S (PDB ID: 6LUD). The

docking results were compared with standard EGFR inhibitors Gefitinib, Afatinib, and Osimertinib. The binding free energies (ΔG) revealed that the synthesized compounds demonstrated strong interactions with the active sites of all EGFR variants. Among the compounds, **Riv** exhibited the highest binding affinity across all variants, with ΔG values of

-12.0, -7.5, and -10.4 kcal/mol, respectively, significantly outperforming the standard inhibitors. Compounds such as **Riii**, **Rviii**, **Rix**, and **Rx** also displayed excellent binding to the drug-resistant EGFRL858R/T790M/C797S mutant, with ΔG values ranging from -10.2 to -10.4 kcal/mol, indicating their potential to overcome resistance associated with the C797S mutation. These findings highlight the importance of the structural features introduced in the synthesized compounds, particularly the variations in R₁ and R₂ substituents, in enhancing their interaction with EGFR. The superior binding affinities, especially against the drug-resistant mutants, underscore the potential of these compounds as promising candidates for further development as EGFR inhibitors.

**Table S1**: Docking score of titled compounds

| **Compound** | **4I23 (EGFRWT)** | **3W2O (EGFRT790M/L858R)** | **6LUD (EGFRL858R/T790M/C797S)** |
| --- | --- | --- | --- |
|  | **ΔG (kcal mol^−1^)** | | |
| **Ri** | -9.6 | -7.8 | -10.0 |
| **Rii** | -9.9 | -9.8 | -10.3 |
| **Riii** | -10.8 | -8.0 | -10.3 |
| **Riv** | -12.0 | -7.5 | -10.4 |
| **Rv** | -9.7 | -7.9 | -10.1 |
| **Rvi** | -9.2 | -7.0 | -9.5 |
| **Rvii** | -9.9 | -7.0 | -9.6 |
| **Rviii** | -10.4 | -7.6 | -10.3 |
| **Rix** | -10.5 | -8.7 | -10.2 |
| **Rx** | -10.6 | -8.8 | -10.1 |
| **Rxi** | -10.2 | -8.6 | -9.9 |
| **Rxii** | -9.8 | -9.0 | -9.5 |
| **Gefitinib** | -9.4 | -8.5 | -7.4 |
| **Afatinib** | -9.0 | -8.9 | -7.6 |
| **Osimertinib** | -8.2 | -8.6 | -7.8 |

- **^1^H and ^13^Cof title compound**

**^1^H of compound Ri**: *N-(4-(4-cyclohexylphenyl)-6-(4-methoxyphenyl)pyrimidin-2-yl)benzamide*.

**^13^C of compound Ri**: *N-(4-(4-cyclohexylphenyl)-6-(4-methoxyphenyl)pyrimidin-2-yl)benzamide*.

**^1^H of compound Rii:** *N-(4-(4-cyclohexylphenyl)-6-(4-(trifluoromethyl)phenyl)pyrimidin-2-yl)benzamide.*

**^13^C of compound Rii:** *N-(4-(4-cyclohexylphenyl)-6-(4-(trifluoromethyl)phenyl)pyrimidin-2-yl)benzamide.*

**^1^H of compound Riii:** *N-(4-(4-cyclohexylphenyl)-6-(2-fluorophenyl)pyrimidin-2-yl)benzamide.*

**^13^C of compound Riii:** *N-(4-(4-cyclohexylphenyl)-6-(2-fluorophenyl)pyrimidin-2-yl)benzamide.*

**^1^H of compound Riv:** *N-(4-(4-cyclohexylphenyl)-6-(3,4-dichlorophenyl)pyrimidin-2-yl)benzamide*.

**^13^C of compound Riv:** *N-(4-(4-cyclohexylphenyl)-6-(3,4-dichlorophenyl)pyrimidin-2-yl)benzamide*.

**^1^H of compound Rviii:** *N-(4-(4-cyclohexylphenyl)-6-(4-(trifluoromethyl)phenyl)pyrimidin-2-yl)cinnamamide*.

**^13^C of compound Rviii:** *N-(4-(4-cyclohexylphenyl)-6-(4-(trifluoromethyl)phenyl)pyrimidin-2-yl)cinnamamide*.

**^13^C of compound Rix:** *N-(4-(4-cyclohexylphenyl)-6-(2-fluorophenyl)pyrimidin-2-yl)cinnamamide.*

**^1^H of compound Rx:** *N-(4-(4-cyclohexylphenyl)-6-(3,4-dichlorophenyl)pyrimidin-2-yl)cinnamamide*.

**^13^C of compound Rx:** *N-(4-(4-cyclohexylphenyl)-6-(3,4-dichlorophenyl)pyrimidin-2-yl)cinnamamide*.

**MS of title compounds**

**ESI-MS Ri:** *N-(4-(4-cyclohexylphenyl)-6-(4-methoxyphenyl)pyrimidin-2-yl)benzamide*.

**ESI-MS Rii:** *N-(4-(4-cyclohexylphenyl)-6-(4-(trifluoromethyl)phenyl)pyrimidin-2-yl)benzamide*.

**ESI-MS Riii:** *N-(4-(4-cyclohexylphenyl)-6-(2-fluorophenyl)pyrimidin-2-yl)benzamide*.

**HR-MS Riv:** *N-(4-(4-cyclohexylphenyl)-6-(3,4-dichlorophenyl)pyrimidin-2-yl)benzamide.*

**HR-MS Rviii:** *N-(4-(4-cyclohexylphenyl)-6-(4-(trifluoromethyl)phenyl)pyrimidin-2-yl)cinnamamide.*

**ESI-MS Rix:** *N-(4-(4-cyclohexylphenyl)-6-(2-fluorophenyl)pyrimidin-2-yl)cinnamamide*.

HPLC purity data of **Riv**: *N-(4-(4-cyclohexylphenyl)-6-(3,4-dichlorophenyl)pyrimidin-2-yl)benzamide.*
